# Exploring precision treatments in immune‐mediated inflammatory diseases: Harnessing the infinite potential of nucleic acid delivery

**DOI:** 10.1002/EXP.20230165

**Published:** 2024-05-24

**Authors:** Lingxiao Xu, Zhenxuan Shao, Xia Fang, Zengfeng Xin, Shenzhi Zhao, Hongbo Zhang, Yu Zhang, Wenbiao Zheng, Xiaohua Yu, Zengjie Zhang, Lingling Sun

**Affiliations:** ^1^ Department of Orthopedic Surgery The Second Affiliated Hospital Zhejiang University School of Medicine Hangzhou China; ^2^ Orthopedics Research Institute of Zhejiang University Zhejiang University School of Medicine Hangzhou China; ^3^ Key Laboratory of Motor System Disease Research and Precision Therapy of Zhejiang Province Zhejiang University School of Medicine Hangzhou China; ^4^ Clinical Research Center of Motor System Disease of Zhejiang Province Zhejiang University School of Medicine Hangzhou China; ^5^ Department of Plastic Surgery The Second Affiliated Hospital Zhejiang University School of Medicine Hangzhou China; ^6^ Pharmaceutical Sciences Laboratory Abo Akademi University Turku Finland; ^7^ Department of Orthopedics Taizhou Municipal Hospital Taizhou China

**Keywords:** delivery systems, immune‐mediated inflammatory diseases, inflammatory bowel disease, nucleic acid therapeutics, psoriasis, rheumatoid arthritis

## Abstract

Immune‐mediated inflammatory diseases (IMIDs) impose an immeasurable burden on individuals and society. While the conventional use of immunosuppressants and disease‐modifying drugs has provided partial relief and control, their inevitable side effects and limited efficacy cast a shadow over finding a cure. Promising nucleic acid drugs have shown the potential to exert precise effects at the molecular level, with different classes of nucleic acids having regulatory functions through varying mechanisms. For the better delivery of nucleic acids, safe and effective viral vectors and non‐viral delivery systems (including liposomes, polymers, etc.) have been intensively explored. Herein, after describing a range of nucleic acid categories and vectors, we focus on the application of therapeutic nucleic acid delivery in various IMIDs, including rheumatoid arthritis, inflammatory bowel disease, psoriasis, multiple sclerosis, asthma, ankylosing spondylitis, systemic lupus erythematosus, and uveitis. Molecules implicated in inflammation and immune dysregulation are abnormally expressed in a series of IMIDs, and their meticulous modulation through nucleic acid therapy results in varying degrees of remission and improvement of these diseases. By synthesizing findings centered on specific molecular targets, this review delivers a systematic elucidation and perspective towards advancing and utilization of nucleic acid therapeutics for managing IMIDs.

## Graphical abstract

Within this review, an in‐depth exploration of therapeutic nucleic acids and their delivery mechanisms in immune‐mediated inflammatory diseases (IMIDs) is presented. The review encapsulates key elements such as different types of nucleic acid molecules, delivery systems for nucleic acids, and the therapeutic use of nucleic acid delivery for IMIDs. Furthermore, the review delves into the potential opportunities and obstacles in the development and utilization of nucleic acid therapeutics within the realm of IMIDs.

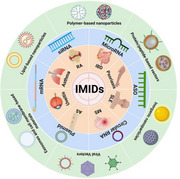

## 1 INTRODUCTION

Immune‐mediated inflammatory diseases (IMIDs) constitute a wide range of chronic inflammatory and autoimmune disorders with shared common inflammatory pathways.^[^ ^1^ ^]^ The multifactorial etiology of these intricate conditions may involve environmental, microbial, immune, and genetic factors, culminating in immune dysregulation and heightened inflammation within the affected organs.^[^ ^2^ ^]^ IMIDs have a high prevalence, impacting approximately 2–5% of the global population.^[^ ^3^ ^]^ Common IMIDs include rheumatoid arthritis (RA), inflammatory bowel disease (IBD), psoriasis, multiple sclerosis (MS), asthma, ankylosing spondylitis (AS), systemic lupus erythematosus (SLE), and uveitis.^[^ ^1^, ^4^ ^]^ In addition, IMIDs often coexist with complications such as cardiovascular diseases, metabolic and skeletal disorders or cognitive impairments, posing significant challenges to disease management.^[^ ^1^ ^]^ Drawing insights from pivotal lessons of the last four decades, the paradigm of IMID treatment has progressively transitioned from broad‐spectrum immunosuppressants to finely tuned, high‐specificity molecular drugs.^[^ ^1^, ^5^ ^]^

Nucleic acid‐based therapies present a promising avenue for treating IMIDs due to their ability to modulate affected genes and alter specific protein expression associated with human diseases, offering several benefits such as precise disease treatment, sustained effects, and personalized medicine potential.^[^ ^6^ ^]^ The various nucleic acid classes encompass plasmids, small interfering RNAs (siRNAs), microRNAs (miRNAs), messenger RNAs (mRNAs), circular RNAs (circRNAs), and antisense oligonucleotides (ASOs), each exhibiting distinct characteristics.^[^ ^7^ ^]^ Plasmids are primarily used to load and express the selected genes. Messenger RNAs serve as templates for the synthesis of specific proteins used for gene therapy or vaccine development. Small interfering RNAs specifically bind to and degrade the mRNA of target genes, whereas miRNA‐mediated mRNA degradation is generally non‐specific. Circular RNAs possess closed‐loop structures that impart high stability to them, and they actively engage in diverse physiological and pathological processes. Through base‐complementary pairing, ASOs play diverse roles in the regulation of gene transcription and translation. Nevertheless, naked nucleic acid drugs are faced with hurdles such as nonspecific in vivo distribution, limited cellular uptake, and rapid clearance, posing challenges in achieving desired outcomes via direct administration.^[^ ^8^ ^]^

For safe and efficient delivery of nucleic acids, both viral and non‐viral based delivery systems are crucial. These aim to safeguard nucleic acids against degradation, optimize their delivery to target cells, and minimize interactions with non‐target cells. Among the earliest biopharmaceutical delivery technologies, virus vectors have been extensively studied for their remarkable transduction efficiency. Over decades of intricate refinement, gene therapy utilizing viral vectors has showcased promising clinical outcomes. Several viral gene therapy products have gained approval for treating conditions such as cancer, genetic disorders, and infectious diseases.^[^ ^9^ ^]^ Another major domain in carrier research explores non‐viral vectors.^[^ ^9^, ^10^ ^]^ Advancements in the development of synthetic materials for encapsulating nucleic acid drugs, including polymer and lipid‐based nanoparticles (NPs), exosomes and membrane‐based nanomaterials, inorganic NPs, and protein/peptide‐based vectors, have invigorated research into non‐viral‐based delivery systems. The value and rapid translational potential of non‐viral delivery systems have been demonstrated by the tremendous success of vaccines from Pfizer‐BioNTech (BNT162b2, also known as Comirnaty) and Moderna (mRNA‐1273, also known as Spikevax).^[^ ^9^ ^]^

**FIGURE 1:**
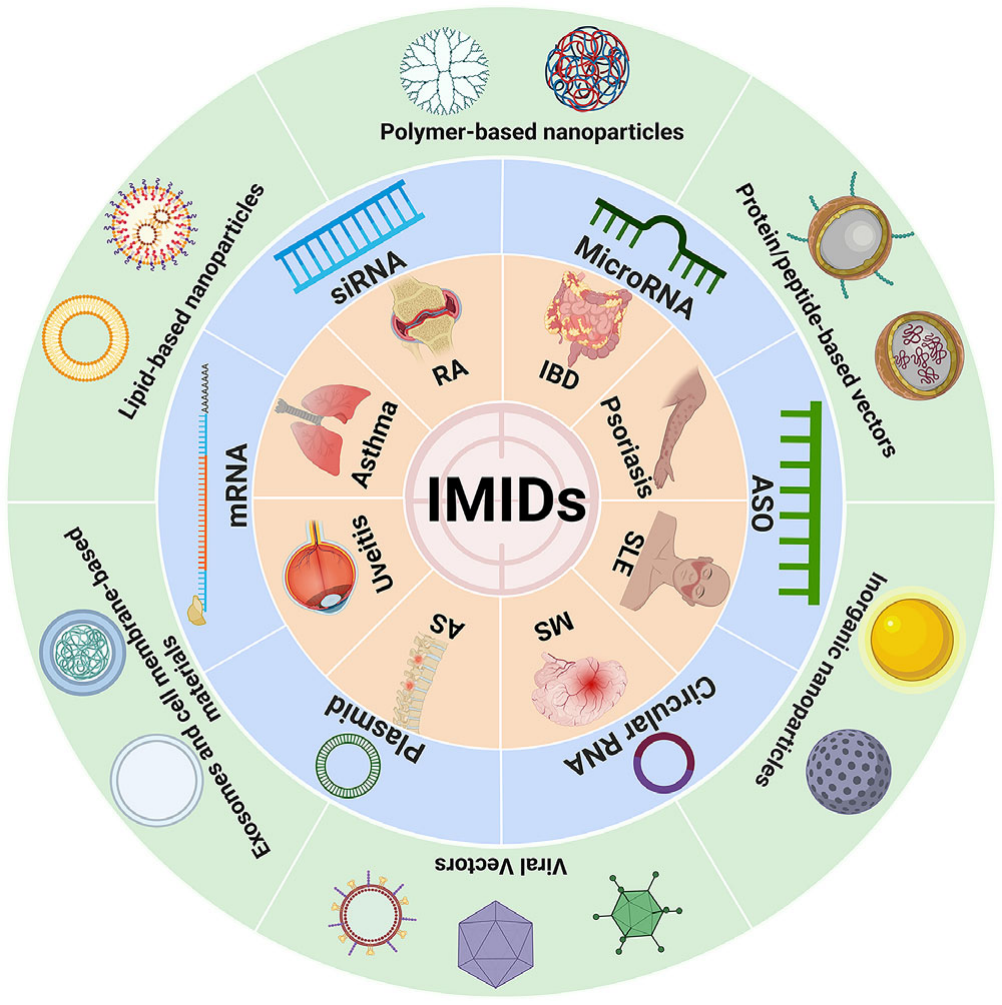
Overview of the structure and content of this review. RA, rheumatoid arthritis; IBD, inflammatory bowel disease; MS, multiple sclerosis; AS, ankylosing spondylitis; SLE, systemic lupus erythematosus; IMIDs, immune‐mediated inflammatory diseases; siRNA, small interfering RNA; ASO, antisense oligonucleotide.

In this review, following the introduction of common nucleic acid classes and both viral and non‐viral carriers used for treatment, we summarize the relevant research based on chosen nucleic acid targets in delivery and further outline the corresponding roles and effects of these therapeutic strategies. Thus, we offer insights into the future application of nucleic therapies for IMIDs (Figure 1). We believe that this summary of our current understanding of the topic will aid in the design of more efficient and safer nucleic acid drugs to treat IMIDs.

## 2 THERAPEUTIC NUCLEIC ACIDS

Nucleic acid drugs exert therapeutic effects by manipulating gene expression or altering the biological properties of targeted living cells. Due to their ability to selectively bind with the human or pathogen transcriptome, they are attractive for traditionally undruggable targets. In contrast to conventional small‐molecule drugs and antibody treatments, nucleic acid drug development is more efficient and highly specific. The nucleic acid therapy market, valued at $4.1 billion in 2021, is projected to reach $12.2 billion by 2031, with an impressive compound annual growth rate of 11.6% from 2022 to 2031 (alliedmarketresearch.com A17089). This section provides a comparison of commonly used therapeutic nucleic acids, as detailed in Table 1.

**TABLE 1:** Comparisons of several properties of the different nucleic acid classes.

	Plasmid DNA	Messenger RNA	Small interfering RNA	MicroRNA	Antisense oligonucleotide	Circular RNA
Abbreviation	pDNA	mRNA	siRNA	miRNA	ASO	circRNA
Structure	Double strand DNA	single‐stranded RNA	Double strand RNA	Double strand RNA	Single strand DNA/RNA	Single strand RNA
Length range (mer)	5000–10,000	1000–2000	20−25	17−25	12−30	Extremely varied range
Action site	Intracellular (nucleus)	Intracellular (cytoplasm)	Intracellular (cytoplasm)	Intracellular (cytoplasm)	Intracellular (nucleus, cytoplasm)	Intracellular (nucleus, cytoplasm)
Target	/	/	mRNA	mRNA	mRNA; pre‐mRNA; miRNA	miRNA; protein
Functions	Loading and expression of desired genes	Translation of proteins/peptides	mRNA cleavage	mRNA degradation	mRNA degradation; Translational inhibition; Splicing regulation; miRNA inhibition	microRNA sponge; Protein sponge; Regulation of gene expression; Translation
Advantages	Low genome integration risk; Low immunogenicity; High stability; Easy transportation and storage	Rapid polypeptide/protein synthesis; No risk of genomic integration; Short development cycle	High specificity; Easy synthesis; High activity; Low immunogenicity	Dual effects on mRNA expression; Regulating multiple disease pathways	High specificity; Easy synthesis; Versatile functionality; Easy delivery; Low immunogenicity	High stability; Low immunogenicity;
Disadvantages	Low transfection efficiency; Low target protein expression level	Immunogenicity; Difficulty in targeted delivery; Strict storage and transportation conditions	Off‐target effect	Low specificity	Off‐target effect	/
Application	Neuvasculgen; Collategene; ZyCoV‐D	BNT162b2; BNT111; mRNA‐1647	Vutrisiran; Inclisiran; QPI‐1002	MRG‐201; MRG‐106	Fomivirsen; Casimersen	VFLIP‐X

### 2.1 Plasmid

**FIGURE 2:**
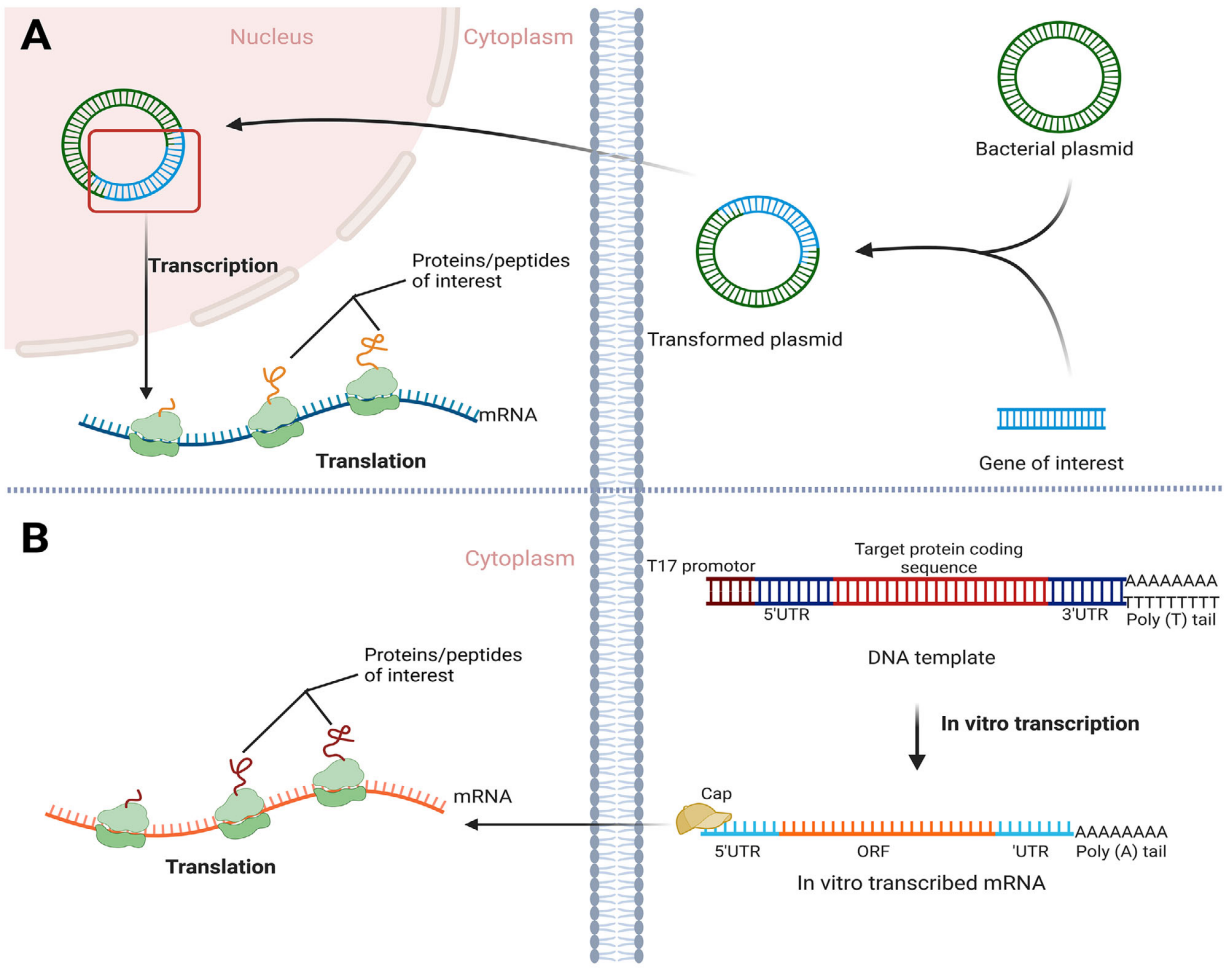
Comparison of differences in the design and mechanism of action between plasmid and mRNA. Plasmids typically need to be delivered to the nucleus to function, while mRNA can be translated to the target proteins in the cytoplasm.

Plasmid‐based drugs are gene therapy products that utilize plasmids for the direct delivery of genetic information, including genes encoding therapeutic proteins, RNA, or antigens, to target cells in patients (Figure 2A and Table 1).^[^ ^11^ ^]^ Plasmids are independent genetic components found in the cytoplasm, separate from the chromosomes. They consist mostly of circular, double‐stranded DNA molecules, typically ranging from 5000 to 10,000 base pairs in length.^[^ ^11a^ ^]^ Plasmids can exist stably and replicate autonomously outside of the chromosomes, or under certain conditions, they can integrate reversibly into host chromosomes during replication.^[^ ^12^ ^]^

The development process for plasmid drugs involves several key stages, including recombinant plasmid construction, large‐scale production, and rigorous quality control. Plasmid‐based drugs offer notable advantages, primarily characterized by their elevated safety profile with minimal risk of gene integration and a reduced likelihood of immunogenicity issues. In comparison to mRNA, plasmid drugs are known for their ease of storage and prolonged stability. Nevertheless, plasmids have a relatively shorter track record in therapeutic applications compared to other biopharmaceuticals, and they encounter ongoing technical hurdles in the areas of process development and industrialization. In addition, low transfection efficiency and restricted target protein expression levels persist as major challenges impeding the progress of plasmid drugs.^[^ ^13^ ^]^

In 2011, Neuvasculgen, a plasmid‐based drug containing plasmids encoding vascular endothelial growth factor (VEGF165), received approval and was introduced to the market in Russia for the treatment of peripheral arterial occlusive disease caused by atherosclerosis (genetherapynet.com HSCI Receives Approval To Market Neovasculgen—The First Russian Gene Therapy Drug For Treatment Of Peripheral Arterial Disease). In 2019, Collategene, another plasmid drug developed by the Japanese company AnGes, containing recombinant human hepatocyte growth factor (HGF), received conditional marketing approval in Japan.^[^ ^14^ ^]^ Collategene is used for the treatment of obstructive arteriosclerosis and thromboangiitis obliterans. In 2021, ZyCoV‐D, a plasmid vaccine developed by Cadila Pharmaceuticals in India, received emergency use authorization for the prevention of COVID‐19 infection in individuals aged 12 and above.^[^ ^15^ ^]^ ZyCoV‐D is the world's first approved plasmid vaccine for human use, marking a significant milestone in the field of plasmid vaccines for various diseases.

### 2.2 Messenger RNA (mRNA)

Messenger RNAs are single‐stranded RNA molecules ranging from 1000 to 2000 nucleotides and can accomplish gene information transmission within the cytoplasm (Figure 2B, Table 1).^[^ ^16^ ^]^ They serve as carriers for expressing infectious agents, cancer antigens, gene editing components, or disease‐related therapeutic proteins, enabling a wide range of clinical applications, including vaccines, gene editing, and protein therapy.^[^ ^16^, ^17^ ^]^

In vitro transcription (IVT) of mRNAs is performed using linearized plasmid templates or PCR templates, which require at least one promoter and the corresponding mRNA coding sequence. The process of obtaining mRNA involves capping, typically achieved through two methods: cotranscriptional capping and posttranscriptional capping.^[^ ^18^ ^]^ The poly(A) tail of IVT mRNA is typically encoded within the DNA template or enzymatically added to the IVT mRNA. Furthermore, purification of IVT mRNAs is necessary, involving the removal of immunogenic impurities, free nucleotides, short mRNAs, and DNA templates. mRNA‐based therapies offer advantages such as short development cycles, rapid rates of polypeptide/protein synthesis, and no risk of genomic integration.^[^ ^19^ ^]^ However, mRNAs exhibit a certain level of immunogenicity, making them susceptible to allergic reactions. Additionally, challenges in efficient delivery and the need for stringent storage and transportation conditions remain as constraints that must be addressed.

Two highly effective mRNA COVID‐19 vaccines, BNT162b2 and mRNA‐1273, were successfully developed and widely deployed at unprecedented speed, showcasing the immense potential of mRNA‐based therapy.^[^ ^20^ ^]^ In addition to COVID‐19 vaccines, significant progress has been made in recent years in the development of mRNA vaccines targeting other infectious diseases and cancer. For instance, Moderna's mRNA‐1647 vaccine (cytomegalovirus vaccine) and mRNA‐1345 vaccine (respiratory syncytial virus vaccine) are currently undergoing phase III clinical trials.^[^ ^21^ ^]^ mRNA vaccines for melanoma, such as BNT111 and mRNA‐4157 developed by BioNTech and Moderna, respectively, have entered phase I and phase II clinical trials.^[^ ^22^ ^]^ Furthermore, mRNA therapeutics have the ability to achieve overexpression, activation, inhibition, and gene deletion of target genes by expressing various proteins. For example, in the context of CAR‐T cell therapy, patients received LNP‐delivered mRNA encoding CAR‐T target antigens, resulting in the generation of functional CAR‐T cells that can be employed in the treatment of tumors or cardiac fibrosis.^[^ ^23^ ^]^ In nonhuman primates, a single‐dose LNP‐delivered mRNA therapy encoding an adenine base editor (ABE), developed by Verve Therapeutics, almost completely suppressed PCSK9 expression in the liver (clinicaltrials.gov NCT05398029). NTLA‐2001, a gene editing therapy based on Cas9 mRNA and sgRNA, effectively reduced serum thyroxine protein levels in human clinical trials.^[^ ^24^ ^]^

### 2.3 Small interfering RNA (siRNA)

Small interfering RNAs are naturally occurring or synthetically produced double‐stranded RNA molecules with a length of approximately 20−25 base pairs. Through the RNA interference (RNAi) mechanism, siRNA has been shown to effectively reduce gene expression in human cells.^[^ ^25^ ^]^ Given that many diseases affecting the population involve some form of abnormal gene regulation, siRNA has emerged as an appealing drug for the treatment of various diseases, including cancer, viral infections, genetic disorders, and metabolic disorders.^[^ ^26^ ^]^

**FIGURE 3:**
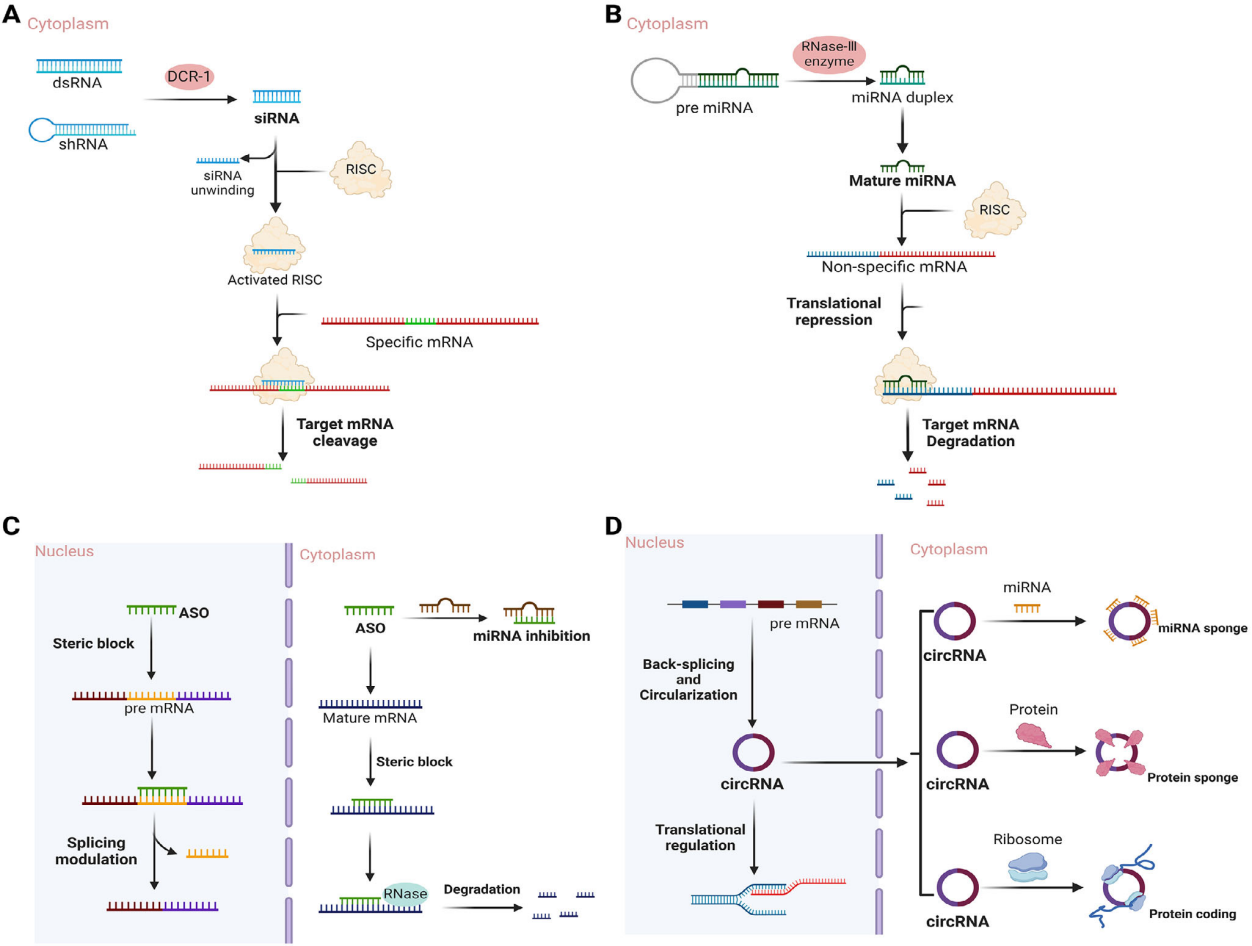
(A) and (B) respectively demonstrate the inhibition of mRNA by small interfering RNA (siRNA) and microRNA (miRNA). (C) and (D) show the multifunctionality of antisense oligonucleotide (ASO) and circular RNA (circRNA). (C) ASOs can selectively correct the missplicing process through exon skipping and inclusion (i); ASOs can bind the target mRNA and recruit RNase H to achieve degradation of the target mRNA (ii); ASOs can bind microRNA and inhibit its function, thereby increasing the expression of its target mRNA (iii). (D) circRNAs serve as miRNA sponge to regulate the expression of target genes (i); circRNAs can directly accumulate in the parental transcribed region to regulate the expression of downstream genes (ii); circRNAs can sponge proteins by binding to them and thus indirectly regulate their functions (iii); circRNAs can translate into peptides or proteins (iv).

siRNA mediated‐RNAi is initiated in the cytoplasm by the ribonuclease Dicer, which cleaves longer double‐stranded RNA (dsRNA) or short hairpin RNA (shRNA) into mature siRNA molecules (Figure 3A). Mature siRNAs are then integrated into the RNA‐induced silencing complex (RISC), which comprises proteins such as Dicer and Ago‐2.^[^ ^27^ ^]^ The siRNA strands are separated, with the guide strand retained as the antisense strand and the passenger strand released, forming the mature RISC. The guide strand aligns the complex with the target mRNA sequence, triggering its cleavage via the Ago‐2 endonuclease.^[^ ^27^ ^]^ siRNA drugs provide high efficiency and specificity in gene silencing, and they are often easy to synthesize. However, unintended gene silencing caused by off‐target effects can potentially result in toxicity and adverse effects.

The therapeutic application of siRNA drugs has progressively expanded to encompass a diverse range of diseases and metabolic abnormalities. Vutrisiran and Patisiran are two FDA‐approved siRNA drugs for the treatment of hereditary transthyretin‐mediated amyloidosis (hATTR).^[^ ^28^ ^]^ Both drugs target the TTR gene and reduce its expression, preventing the formation of amyloid deposits. Inclisiran inhibits PCSK9 mRNA and thus lowers low‐density lipoprotein cholesterol levels.^[^ ^29^ ^]^ Givosiran utilizes the ESC‐GalNAc delivery platform and targets the ALAS1 gene to reduce the accumulation of neurotoxic heme intermediates, including aminolevulinic acid (ALA) and porphobilinogen (PBG).^[^ ^30^ ^]^ QPI‐1002, the first systemically administered siRNA drug to enter human clinical trials, aims to inhibit the expression of the proapoptotic gene p53, thereby protecting normal cells from acute tissue injury‐induced death.^[^ ^31^ ^]^

### 2.4 MicroRNA (miRNA)

MicroRNAs are endogenous, non‐coding single‐stranded RNA molecules approximately 17–25 nucleotides in length.^[^ ^32^ ^]^ They regulate gene expression at the post‐transcriptional level and play crucial roles in various biological processes such as cell proliferation, differentiation, apoptosis, and hematopoiesis.^[^ ^32^ ^]^ It is estimated that mammalian cells contain approximately 5000‐10,000 miRNAs, which regulate the expression of over 60% of protein‐coding genes.^[^ ^33^ ^]^

The biogenesis of miRNAs involves a series of intricate steps (Figure 3B).^[^ ^32^ ^]^ It commences with transcription into pri‐miRNA, which is subsequently processed by Drosha, exported from the nucleus via Exportin 5 and further cleaved by Dicer. Afterward, one strand is selected and the mature miRNA is incorporated into the RNA‐induced silencing complex (RISC), where it plays a pivotal role in the regulation of mRNAs. Typically, miRNAs are known to inhibit mRNA translation (Figure 3B); however, they can also upregulate the expression levels of target mRNAs.^[^ ^34^ ^]^ Additionally, miRNAs within the nucleus can activate gene transcription by interacting with long non‐coding RNAs.^[^ ^34^, ^35^ ^]^ MiRNAs can simultaneously regulate one or multiple complete disease pathways.^[^ ^36^ ^]^ However, this may also imply relatively low specificity.

MiRNA‐based therapeutic strategies primarily employ miRNA mimics to supplement corresponding biological functions or miRNA inhibitors to suppress disease‐promoting miRNA. MRG‐201 (Remlarsen), developed by miRagen Therapeutics, is a fibrosis‐inhibiting drug that mimics miR‐29b, which is currently in a phase 2 clinical trial.^[^ ^34^ ^]^ By inhibiting fibrosis development in skin wounds, it holds potential to provent fibrotic scar formation and skin fibrosis. MRG‐106 (Cobomarsen) is an inhibitor of miR‐155 and is currently undergoing a phase 2 clinical trial.^[^ ^37^ ^]^ miR‐155, which regulates the differentiation and proliferation of blood and lymphatic cells, demonstrates high expression levels in various hematologic malignancies, including diffuse large B‐cell lymphoma.^[^ ^37^ ^]^ Therefore, inhibiting miR‐155 is an important therapeutic strategy for hematologic tumors, and promising results have been observed in animal experiments where it demonstrated the potential to reduce tumor volume.

### 2.5 Antisense oligonucleotides (ASOs)

Antisense oligonucleotides are synthetic polymers typically ranging from 12 to 30 nucleotides in length, that mimic the behavior of DNA/RNA and engage target RNA through Watson‐Crick base pairing.^[^ ^38^ ^]^ Recently, the mechanisms underlying ASO functions have gained further clarity (Figure 3C). They possess the ability to impact mRNA maturation by impeding cap formation or 3′‐end polyadenylation. ASOs can selectively govern pre‐mRNA splicing, rectifying errors in the process.^[^ ^39^ ^]^ Upon binding to target mRNA, they recruit RNase H or Argonaute 2 (Ago 2) to degrade or silence mRNA.^[^ ^40^ ^]^ ASOs can also disrupt RNA‐ribosome interactions, halting translation.^[^ ^41^ ^]^ Certain ASOs can even modify RNA structures that inhibit translation, obstruct upstream AUG codons, or bind to miRNAs, thus enhancing protein translation.^[^ ^42^ ^]^ Challenges such as limited cellular uptake,^[^ ^43^ ^]^ potential off‐target effects,^[^ ^44^ ^]^ and a short half‐life have led to the exploration of stability‐enhancing modifications of ASOs, including phosphorothioate, phosphoramidate, and locked nucleic acids.^[^ ^45^ ^]^ These structural modifications bestow ASOs with supplementary attributes, such as facilitating their interaction with serum proteins, thereby prolonging serum half‐life; augmenting cellular uptake; enhancing stability and even obviating the requirement for carrier delivery; intensifying drug activity; and diminishing non‐specific protein binding to mitigate toxicity.^[^ ^46^ ^]^ The complementary base pairing enables ASOs to have relatively high specificity. Additionally, ASOs boast low immunogenicity and ease of synthesis. However, the application of ASO may be accompanied by off‐target effects.

The discovery of the action mechanism of ASO has spurred research institutions and pharmaceutical companies to explore their potential clinical applications. The first approved ASO drug, Fomivirsen, was used for treating cytomegalovirus retinitis in AIDS patients (nature.com d42859‐019‐00080‐6). Additionally, eight other ASO drugs have been approved for indications such as spinal muscular atrophy, familial hypercholesterolemia, and transthyretin amyloidosis.^[^ ^47^ ^]^ Casimersen is the latest drug approved for treating Duchenne muscular dystrophy patients with exon 45 skipping.^[^ ^48^ ^]^ Currently, over 50 ASO drugs are in clinical development worldwide, expanding their indications from rare diseases to common conditions such as hepatitis B infection, obesity, leukemia, and malignancies like non‐small cell lung cancer and pancreatic cancer.

### 2.6 Circular RNA (circRNA)

Circular RNAs are a unique class of non‐coding RNAs widely present in eukaryotic cells, featuring highly variable lengths and occasional evolutionary conservation.^[^ ^49^ ^]^ Their distinctive closed‐loop structure yields exceptional stability, making them a promising avenue for therapeutic nucleic acid interventions.^[^ ^50^ ^]^ Once considered splicing errors or byproducts of intron ‘lasso’ structures, circRNAs are now recognized as functionally significant entities. Leveraging their stability and tissue specificity, they actively participate in a spectrum of biological processes.^[^ ^51^ ^]^ CircRNAs influence physiological and pathological processes through distinct mechanisms (Figure 3D). Initially, they contain miRNA binding sites, allowing them to interact with miRNAs and inhibit their binding to the 3′‐UTR region of target mRNA, thus acting as miRNA sponges and controlling mRNA translation. Moreover, they can bind to diverse proteins, effectively acting as protein sponges, leading to the formation of circRNA‐protein complexes (circRNPs). These subsequently modulate protein function, subcellular localization, or target gene expression.^[^ ^52^ ^]^ In addition, a subset of circRNAs, particularly those located in the cell nucleus like ciRNAs and EIciRNAs, may play a role in the transcriptional regulation of parent genes.^[^ ^52^ ^]^ Beyond their RNA‐based regulatory functions, there is substantial evidence suggesting that circRNAs can undergo translation to produce proteins or peptides.^[^ ^53^ ^]^ CircRNAs hold promise for the treatment of various refractory diseases, including viral infections, tumors, pathogenic bacterial infections, autoimmune diseases, and metabolic disorders.^[^ ^54^ ^]^ Currently, circRNAs have been explored in several SARS‐CoV‐2 vaccines. For instance, circRNA‐based vaccine VFLIP‐X can induce robust neutralizing antibody responses against various SARS‐CoV‐2 variants in mice.^[^ ^55^ ^]^ Furthermore, in a study investigating the therapeutic potential of circRNA vaccines in melanoma, significant inhibition of tumor metastasis was observed in mice.^[^ ^56^ ^]^

## 3 DELIVERY SYSTEMS FOR NUCLEIC ACIDS

Non‐specific distribution, enzymatic degradation, and biological barriers pose significant challenges to the efficacy of direct nucleic acid administration.^[^ ^8^ ^]^ Therefore, it is necessary to select, design, and apply suitable carriers for the effective delivery of these nucleic acids. Virus vectors were among the earliest biopharmaceutical delivery technologies, which have been extensively studied for their remarkable transduction efficiency. Over decades of intricate refinement, gene therapy utilizing viral vectors has showcased excellent clinical outcomes. Several viral gene therapy products have gained approval for treating conditions such as cancer, genetic disorders, and infectious diseases.^[^ ^57^ ^]^ While viral vectors offer high gene transfection efficiency, they come with the risks of genomic contamination and immunogenicity. Therefore, recent efforts in treating IMIDs have primarily focused on non‐viral carriers with increased safety and efficacy (Table 3). This section provides an overview of commonly employed viral and non‐viral nucleic acid delivery systems.

### 3.1 Viral vectors

A viral vector is typically characterized by three essential components. The protein capsid and/or envelope surround the genetic payload, determining the vector's host tissue or cell tropism and antigen recognition. When expressed in cells, the transgene imparts the desired effect. The “regulatory cassette,” comprising enhancer, promoter, and auxiliary elements, governs stable or transient somatic expression of the transgene, either as an episome or a chromosomal integrant. In addition, modifications are applied to viral vector genomes, rendering them replication‐defective and thus bolstering safety. Clinical studies have explored five major classes of viruses as vectors, encompassing retrovirus, adenovirus (Ad), adeno‐associated virus, lentivirus, and herpes simplex virus (HSV).^[^ ^58^ ^]^ While retroviruses have fallen out of common clinical use due to their propensity to transduce only dividing cells and the relative high risk of random insertion mutations during host chromosome integration,^[^ ^59^ ^]^ carriers derived from HSV‐1 exhibit the ability to infect non‐dividing cells and replicate within them.^[^ ^60^ ^]^ However, challenges in producing high‐titer vectors and their potential to induce robust inflammatory responses have curtailed the widespread adoption of HSV. Currently, viral delivery technologies in biomedicine primarily center around AdVs, AAVs, and lentiviral vectors that are recognized for their widespread application in successful gene therapy and regulatory endorsement. Key features of these vectors are illustrated in Table 2.

**TABLE 2:** Features of viral vector systems for nucleic acid delivery.

Viral vector	Viral genome	Packaging Capacity	Integration into host genome	Merits	Disadvantages
AdV	dsDNA	>7.5 kb	No	High infection efficiency; Infection of dividing and nondividing cells; Low host specificity	Potent immunogenicity; Short‐lived transgene expression (1−2 weeks)
AAV	ssDNA	4.7 kb	No (recombinant AAV genome)	Long‐term stable expression; Infection of dividing and nondividing cells; Low level immune response	Limited transgene capacity; Complicated production process; Adverse events associated with high‐dose therapy
Lentiviral vector	RNA	9 kb	Yes	High infection efficiency; Infection of dividing and nondividing cells; Expression of multiple genes from a single vector; Low immunogenicity	Complicated design and construction; Activation of oncogenes; Abnormal splicing; Concerns of biosafety; Potential generation of pathogenic gene (revertant replication‐competent HIV)

**Abbreviations**: AAV, Adeno‐associated viral vector; AdV, Adenoviral vector.

#### 3.1.1 Adenoviral vectors (AdVs) and adeno‐associated viral vectors (AAVs)

**FIGURE 4:**
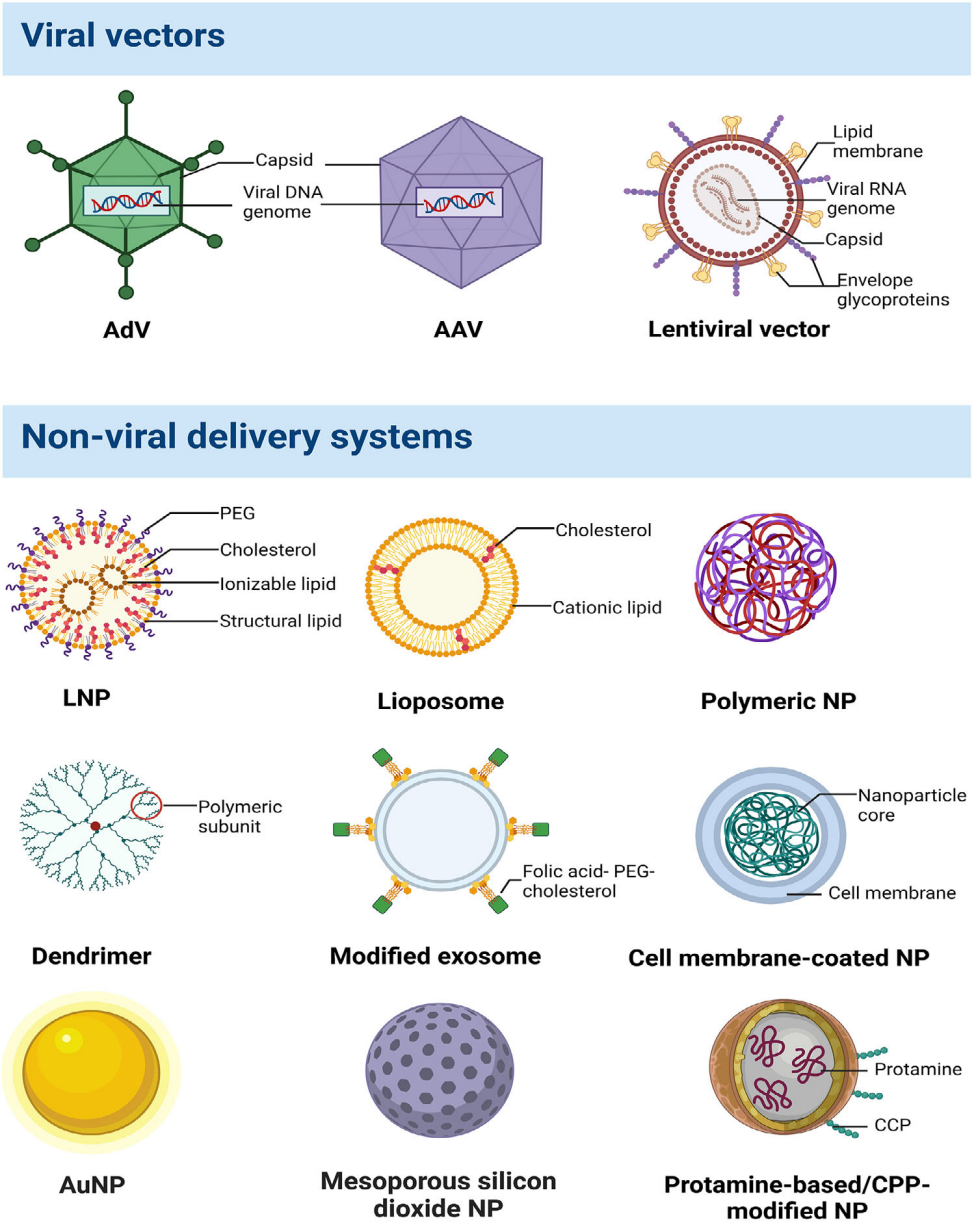
Schematic illustration of different viral and non‐viral carriers. Currently, viral delivery technologies in biomedicine primarily center around AdVs, AAVs, and lentiviral vectors. On the other hand, various non‐viral vectors have received widespread attention, including lipid and polymer‐based nanoparticles, exosomes and membrane‐based nanomaterials, inorganic nanoparticles, and protein/peptide‐based vectors. AdV, adenoviral vector; AAV, adeno‐associated viral vectors; LNP, lipid nanoparticle; CCP, cell‐penetrating peptide.

AdV was among the first viral vectors investigated in the clinic for in vivo gene therapies. Adenovirus (Ad) is a class of double‐stranded DNA viruses with a 34−43 kb genome enclosed in a non‐enveloped icosahedral viral particle (Figure 4). The Ad genome is flanked by two hairpin‐like inverted terminal repeats (ITRs), serving as self‐priming structures that promote primase‐independent DNA replication. The Ad genome comprises five so‐called “early‐phase” genes, E1A, E1B, E2, E3, and E4, which are responsible for viral DNA synthesis and play roles in modulating the expression of host genes.^[^ ^61^ ^]^ The late regions (L1–L5) are transcribed from an alternatively spliced transcript and are generally required for virus assembly and release and the lysis of host cell.^[^ ^62^ ^]^ Once it has reached the cell nucleus, viral DNA predominantly remains epichromosomal and is not incorporated into the host cell genome.^[^ ^63^ ^]^ There are more than 50 Ad serotypes, with Ad5 and Ad26 being most widely used for gene therapy.^[^ ^64^ ^]^ Three generations of AdVs have been engineered to fit different therapeutic applications. The first involves the removal of E1 and E3 units, that makes the vector capable of carrying up to 7.5 kb of foreign DNA.^[^ ^65^ ^]^ The E1 and E4 units are deleted in the second generation of AdVs, significantly reducing immunogenicity.^[^ ^66^ ^]^ All viral genes are deleted in the third generation, referred to as “gutless” or “helper‐dependent” AdVs, which allows the vector to carry more than 30 kb of foreign DNA.^[^ ^67^ ^]^ AdVs boast high transduction efficiency both in quiescent and dividing cells and large transgene capacity, and are free of host specificity.^[^ ^68^ ^]^ Nevertheless, the development of AdVs faces challenges associated with adverse immune and inflammatory responses.^[^ ^69^ ^]^ In addition, the duration of transgene expression is relatively short (1−2 weeks).^[^ ^68b^ ^]^

AAVs have been universally recognized as versatile vectors for gene therapy and have been successfully employed in FDA‐approved drugs and therapeutic clinical trials.^[^ ^70^ ^]^ They consist of a non‐enveloped icosahedral capsid and a 4.7 kb single‐stranded DNA genome housing two distinct open reading frames: Rep and Cap (Figure 4). AAVs clone and carry the desired transgene between the ITR sequences (genome replication and packaging signals) that interact with Rep. Any transgene shorter than 4.7 kb can be inserted into the AAV vector. AAVs encompass 11 natural serotypes and over 100 variants.^[^ ^71^ ^]^ Each serotype exhibits tropism towards a specific tissue, making it suitable for delivering genes to a specific organ. Wild‐type AAVs can integrate into the genome of the host cell,^[^ ^72^ ^]^ while the recombinant AAV genome (rAAV), achieved by gutting the genome and saving the ITR elements, remains in an extrachromosomal form following infection.^[^ ^73^ ^]^ When designing AAV screens, the first consideration is whether to quantify AAV distribution (i.e. whether AAVs reach the cells) or functional delivery (i.e. whether AAVs should reach the cell nucleus or produce target mRNA or proteins). The second aspect to consider is the species dependency of AAV‐mediated delivery. In humanized mice with livers recombinantly engineered from mouse and human cells, the optimal AAV vector for delivery varies between the hosts.^[^ ^74^ ^]^ Compared to other current viral vectors, rAAVs are accepted as the least immunogenic ones, with much less vector‐related toxicity. In contrast to Ad vectors, AAVs are ideal for their lack of transgene immunity when expressing self‐antigens and confer relatively low innate immunity and viral immunity within a broad dose range.^[^ ^73^ ^]^ Moreover, they provide long‐term stable transgenic expression. In order to avoid the activation of Toll‐like receptor 9 (TLR9) by AAVs and the subsequent induction of unnecessary immune reactions, Chan et al. integrated short DNA oligonucleotides that antagonize TLR9 activation directly into the viral genome to reduce immunogenicity.^[^ ^75^ ^]^ However, limitations remain, such as the potential for adverse events under high‐dose AAV therapy and limited vector capacity. In addition, the process of AAV production is relatively complicated.

#### 3.1.2 Lentiviral vectors

Lentiviruses as a subclass of retroviruses, are spherical and enveloped, encapsidating two sense‐strand RNAs bound by nucleocapsid proteins (Figure 4). Reverse transcriptase, integrase, and protease proteins are also contained.^[^ ^69^ ^]^ Taking the lentivirus HIV‐1 as an example, the 9.7 kb HIV‐1 genome is flanked by 5′‐ and 3′‐LTRs, which are integral to viral genome replication. The cis‐acting sequence (psi) is pivotal for signaling viral genome encapsidation. Gag, pol and env are common essential core protein genes. The gag gene encodes the protective capsid and matrix proteins, while the env gene carries information on transmembrane and envelope glycoproteins that dictate the cellular tropism of the virus.^[^ ^76^ ^]^ Reverse transcriptase and integrase are transcribed from the pol gene. Lentiviruses have additional genes called tat and rev to facilitate viral replication. Tat supports transcriptional activation and RNA polymerase II elongation by binding adjacently to the LTR. Rev orchestrates the nuclear export of spliced and un‐spliced viral RNA by binding to a motif in the env gene region. Lentiviruses have an additional set of auxiliary genes (vif, vpr, vpu, and nef), which elevate the viral titer and pathogenesis of the virus.^[^ ^77^ ^]^

Divergent from other retroviruses (e.g. gammaretroviral vectors), lentiviruses exhibit a unique ability to integrate and transduce their genome into non‐dividing cells. These vectors accommodate up to 9 kb of genetic material and facilitate prolonged transgene expression by enabling the efficient integration of foreign genes into the host chromosome. Lentiviral vectors have also proven their potential to express multiple genes from a single vector, satisfying the imperative need for the high‐level expression of multiple genes in the treatment of specific diseases.^[^ ^78^ ^]^ In addition, the immune responses induced by lentiviral vectors vary based on the vector design, often displaying a tendency toward modest reactivity.^[^ ^79^ ^]^

The lentiviral vector system, derived from the HIV‐1 virus and developed over many years, aims to mitigate potential risks associated with virus‐based platforms. A notable safety consideration in designing lentiviral vector systems for gene therapy is the inadvertent generation of replication‐competent original viruses. In contrast to first‐generation HIV‐1‐based vectors that retain most of the viral genome within the trans‐packaging construct, the safer second‐generation lentiviral vectors lack accessory genes (vif, vpr, vpu, and nef) promoting virus proliferation and infection. To limit the formation of unintended replication‐competent original viruses, a third‐generation vector lacking tat and rev regulatory genes in the packaging structure has been developed. The rev gene required for replication is provided as a trans‐package, creating a conditional packaging platform. The third‐generation lentiviral system further removes a portion of the 3′‐LTR retrotransposon (containing the TATA box and transcription factor binding sites), enhancing safety and establishing a self‐inactivating vector packaging system.

Additional modifications (e.g. transcriptional regulatory elements,^[^ ^80^ ^]^ the scaffold attachment region of the interferon‐β gene,^[^ ^81^ ^]^ and the woodchuck hepatitis virus post‐transcriptional regulatory element (WPRE)^[^ ^82^ ^]^) aim to boost the expression and transduction efficiency of lentiviral vectors.

Achieving tissue or cell‐type specificity is a key aspect of designing efficient and safe lentiviral vectors. One such approach involves constructing pseudotypes.^[^ ^83^ ^]^ Pseudotyped lentiviral vectors integrate envelope glycoproteins obtained from other enveloped viruses upon release from the host cell, thereby acquiring the tropism characteristic of the source virus.^[^ ^83^ ^]^ For example, incorporating the neurotropic rabies virus glycoprotein (RVG) as a pseudotype for the lentiviral vector facilitates retro‐axonal and trans‐synaptic propagation, thus amplifying transgene expression within the brain.^[^ ^84^ ^]^ An alternative strategy entails creating tissue‐specific expression vectors, wherein a tissue‐specific promoter ensures limited transgene expression exclusively within the targeted cells. The CD19 gene promoter and immunoglobulin κ light chain promoter have been employed to achieve B‐cell specificity in lentiviral vectors,^[^ ^85^ ^]^ while the CD4 gene promoter has been shown to enable T‐cell targeting.^[^ ^86^ ^]^ The temporal regulation of lentiviral vector expression can be achieved using a tetracycline‐inducible promoter.^[^ ^87^ ^]^

The utilization of third‐generation self‐inactivating lentiviral vectors is instrumental in reducing the risk of insertional mutations.^[^ ^88^ ^]^ Nevertheless, even self‐inactivating vectors with robust promoters and enhancer elements may activate neighboring genes. Mitigating these effects involves incorporating insulator sequences to counteract the impact of trans‐acting enhancers.^[^ ^89^ ^]^ Integration events may further result in the creation of chimeric gene fusions containing both viral and host sequences.^[^ ^90^ ^]^ Finally, lentiviral vectors have been shown to induce abnormal splicing in cellular transcripts.^[^ ^91^ ^]^

### 3.2 Non‐viral delivery systems

#### 3.2.1 Lipids and lipid‐based nanoparticles

Lipids form various structures, including micelles, liposomes, and lipid NPs (LNPs), based on the size ratio between the hydrophilic head group and the hydrophobic tail(s). Unlike liposomes with a singular aqueous core, LNPs boast a multi‐layered core, intricately woven with lipid and nucleic acid contraction loops.^[^ ^92^ ^]^ FDA‐approved LNPs contain variations of four basic components: a cationic or ionizable lipid, cholesterol, a helper lipid (mainly phospholipids), and a poly(ethylene glycol) (PEG)‐lipid (Figure 4).^[^ ^93^ ^]^ Cationic lipids carry a positive charge under physiological conditions, facilitating interactions with negatively charged molecules and making them susceptible to immune cell capture, potentially shortening LNP circulation time.^[^ ^92a^ ^]^ In contrast, ionizable lipids maintain a neutral charge at physiological pH, offering a method to mitigate toxicity and extend LNP circulation half‐life.^[^ ^94^ ^]^ Moreover, ionizable lipids acquire a positive charge in low‐pH environments, driving the encapsulation of negatively charged nucleic acid drugs within LNPs. This also promotes interactions with negatively charged intracellular membranes at lower pH levels, enabling endosomal escape and enhancing delivery efficiency. Notably, DLin‐MC3‐DMA (MC3) stands as the first FDA‐approved ionizable lipid for nucleic acid delivery.^[^ ^95^ ^]^ Riley et al. demonstrated that LNPs composed of MC3 could efficiently deliver mRNA to mouse embryos in the uterus.^[^ ^96^ ^]^ In recent developments, Moderna introduced a range of ionizable lipids, such as Lipid 5, which demonstrated diminished immunostimulatory effects in non‐human primates compared to MC3.^[^ ^97^ ^]^ Serving as the structural foundation of LNPs, phospholipids (e.g. distearoylphosphatidylcholine (DSPC) and dipalmitoylphosphatidylcholine (DPPC)) contribute to the formation of lipid bilayers within endosomes and facilitate the transition from lamellar to hexagonal phases, promoting nucleic acid cytoplasmic release.^[^ ^98^ ^]^ PEG‐lipids enhance LNP stability.^[^ ^99^ ^]^ PEG chains on LNP surfaces create a hydrophilic spatial barrier, promoting LNP self‐assembly and preventing aggregation.^[^ ^99^ ^]^ Moreover, they hinder the binding of non‐specific proteins, including plasma proteins, preventing rapid clearance by the reticuloendothelial system and extending circulation duration.^[^ ^100^ ^]^ Lokugamage et al. indicated that PEG‐lipid deficiency results in unstable, highly dispersed LNPs with increased polydispersity indices, while PEG‐lipid‐containing LNPs improve mRNA delivery to the lungs.^[^ ^101^ ^]^

Functioning as biomimetic structures, liposomes can be crafted from either natural or synthetic phospholipids (Figure 4).^[^ ^102^ ^]^ Cholesterol is commonly incorporated into liposomal formulations to prevent drug leakage by interacting with their alkyl chains, reducing membrane fluidity, and providing stability in vitro and in vivo.^[^ ^103^ ^]^ The presence of cholesterol may also impact the intracellular transport of liposomal phospholipids.^[^ ^104^ ^]^ Depending on the phospholipid composition, liposomes manifest as neutral, cationic, or anionic entities.^[^ ^105^ ^]^ Common neutral phospholipids include phosphatidylcholine, phosphatidic acid, and phosphatidylethanolamine. Anionic phospholipids bear negative charges, such as phosphatidylglycerol and phosphatidylserine, while cationic lipids like *N*‐[1‐(2,3‐dioleyloxy)propyl]‐*N,N,N*‐trimethylammonium chloride (DOTMA) and 1,2‐bis(oleoyloxy)−3‐(trimethylammonium)propane (DOTAP) find extensive use in gene delivery.^[^ ^106^ ^]^ Cationic liposomes exhibit the enhanced loading of negatively charged nucleic acids and easier escape from endosomes, facilitating the release of loaded nucleic acids into the cytoplasm.^[^ ^107^ ^]^ The potential cytotoxicity of cationic lipids with quaternary ammonium groups poses a significant obstacle, which has hindered their clinical translation.^[^ ^108^ ^]^ To address this issue, Schlegel et al. encapsulated cationic liposomes with non‐toxic, biodegradable anionic polymers such as sodium polyacrylate, hyaluronic acid, sodium alginate, and sodium polyglutamate, thus reducing toxicity while enhancing siRNA delivery to liver and lung tissues.^[^ ^109^ ^]^

#### 3.2.2 Polymers and polymer‐based nanoparticles

Numerous non‐viral nucleic acid delivery systems leverage additional polymers and polymer nanoparticles (Table 3). Modifying the polymer properties like charge, degradability and molecular weight significantly shapes how nucleic acids are delivered into cells.^[^ ^110^ ^]^ Natural polymeric materials, including chitosan (CSs), hyaluronic acid (HA), cyclodextrins (CDs), are valued for their biocompatibility, biodegradability, and low toxicity.^[^ ^111^ ^]^ CSs, derived from chitin found in the exoskeletons of crustaceans and the cell walls of fungi through deacetylation, is a promising carrier for nucleic acid delivery due to its strong cationic charge.^[^ ^112^ ^]^ However, CSs face challenges, including limited cell membrane penetration and endosomal escape capabilities. Researchers have addressed these difficulties by attaching low‐molecular‐weight polyethyleneimine (PEI), enhancing transfection efficiency and siRNA silencing efficacy.^[^ ^113^ ^]^ To improve CSs water solubility, some studies performed a Michael addition reaction with 2‐propenamide‐2‐methylpropane sulfonic acid (AMP).^[^ ^114^ ^]^ HA, a natural polysaccharide, is often used with cationic polymers like PEI to reduce interactions with poly‐anionic biomolecules, enhancing stability in physiological conditions.^[^ ^115^ ^]^ HA can also act as a protective shield when coating the surface of polyethylene amine (PEA)/anti‐miR‐155 complexes, reducing interactions with poly‐anionic biomolecules.^[^ ^116^ ^]^ CDs enhance membrane permeability in gene delivery systems and can be modified to improve transfection efficiency.^[^ ^117^ ^]^ Kihara et al. synthesized a series of polyamidoamine (PAMAM) dendrimers with different average α‐cyclodextrin (α‐CD) substitution degrees for the delivery of plasmid. Following the intravenous injection of plasmid complexes into mice, the PAMAM‐α‐CD conjugate exhibited optimal plasmid delivery efficacy at a substitution degree of 2.4.^[^ ^118^ ^]^ Some preliminary research explored nucleic acid delivery systems based on other natural polymers, like quaternized starch for siRNA^[^ ^119^ ^]^ and alginates for plasmid.^[^ ^120^ ^]^

**TABLE 3:** Summarizing research on nucleic acid delivery for treating various IMIDs.

Therapeutic nucleic acid and drugs	Nanoparticle composition	Administration route	Animal model	Mainly regulated cells	Effects	Reference
**RA**						
Plasmid coding TNF‐α shRNA	Cationic polymer PDAPEI synthesized from 2,6‐pyridinedicarboxaldehyde and low‐molecular‐weight PEI	i.v.	CIA mice	Macrophages	Significantly down‐regulating the expression of TNF‐α; Efficiently decreased the severity of arthritis	[173]
TNF‐α siRNA	Chitosan	i.p.	CIA mice	Macrophages	Specific TNF‐α knockdown (≈44%); Downregulated systemic and local inflammation; Arrested joint swelling; Minimal cartilage destruction and inflammatory cell infiltration	[174]
TNF‐α siRNA	PLGA/DOTAP	i.a.	CAIA mice	Macrophages	Silenced TNF‐α expression in vitro; Reduced disease activity, paw scores and joint effusion	[175b]
TNF‐α siRNA	Cationic liposome 2‐(3‐[Bis‐(3‐amino‐propyl)‐amino]‐propylamino)‐N‐ditetradecylcarbamoylme‐thyl‐acetamide)/DOPE	i.v.	CIA mice	Macrophages	Inhibition (50−70%) of articular and systemic TNF‐α secretion; Reduction of IL‐6 and MCP‐1; Increased production of the anti‐inflammatory cytokine IL‐10; Reduced incidence and severity of the disease	[175a]
TNF‐α siRNA	Lipidoid and PLGA	i.a.	Dimethyldioctadecylammonium and human proteoglycan induced arthritis mice	Macrophages	Specifically inhibited expression of TNF‐α; Relieved inflammation; Reduced disease severity and delayed onset of arthritis	[175c]
TNF‐α siRNA	Liposome	i.v.	CIA mice	CD11b^+^ cells (macrophages and neutrophils)	Decreased arthritis severity and TNF‐α mRNA level; Reduced arthritis incidence and clinical arthritis scores	[219]
TNF‐α siRNA	Macrophage membrane vesicles and prussian blue nanoparticles	i.v.	CIA mice	FLSs; Macrophage; inflamed vessels cells	Down‐regulation of TNF‐α and IL‐6 expression; weakened invasiveness of CIA‐FLS; effectively inhibited bone erosion and cartilage destruction; significantly relieved synovial fibrosis, synovial hyperplasia and immune cell infiltration	[202]
TNF‐α siRNA	Chitosan nanocarrier consisting of folate, diethylethylamine and PEG	i.p.	CIA mice	Osteoclasts	Decreased level of TNF‐α; anti‐osteoclastogenesis properties; Decreased articular cartilage destruction and bone loss	[165a]
IL‐1β siRNA	Polymer‐lipidoid hybrid nanoparticle (FS14‐NP) composed of Pluronic F127 and spermidine‐based lipidoid (S14)	i.v.	CAIA mice	Macrophages	Diminished expression of IL‐1β, TNF‐α, MMP‐3, and MMP‐13; Alleviation of bone erosion and cartilage destruction	[177]
Plasmid coding IL‐1 receptor antagonist	NPs made of folic acid and chitosan	i.v.	AIA rats	Osteoclasts	Decreased level of IL‐1β; Regulated markers of osteoblast metabolism; Decreased osteoclast number; Prevented bone damage and inflammation	[165b]
IL‐1β/IL‐6/IL‐18 siRNA	Cationic liposome 2‐(3‐[Bis‐(3‐amino‐propyl)‐amino]‐propylamino)‐N‐ditetradecylcarbamoylme‐thyl‐acetamide)/DOPE	i.v.	CIA mice	Macrophages	Reduced local secretion of IL‐1β, IL‐6 and TNF‐α and increased IL‐10 production; Improved pathological signs of arthritis	[178]
Plasmid coding IL‐10	Non‐condensing alginate‐based NPs modified with tuftsin peptide	i.p.	Heat‐killed Mycobacterium butyricum and incomplete Freund's adjuvant induced arthritis in rats	Macrophages	Macrophage repolarization from M1 to M2; Reduced systemic and joint tissue pro‐inflammatory cytokines (TNF‐α, IL‐1β, and IL‐6) expression; Prevented progression of inflammation and joint damage	[181]
Plasmid coding IL‐10 and BSP	M2 macrophage exosomes	i.v.	CIA mice	Macrophages	Up‐regulated mRNA levels of IL‐10 and IL‐4; Down‐regulated the mRNA levels of IL‐1β and TNF‐α; Reduced inflammatory cells infiltration and bone erosion degree; Inhibited cartilage cells degeneration	[182]
IL‐15Rβ siRNA	PEI	i.v.	AIA rats	Macrophages	Decreased expression of TNF‐α and IL‐1β; Relieved clinical symptoms; Reduced synovial tissue inflammation and cartilage/bone destruction	[180]
cPLA_2_α siRNA	Cationic liposome 2‐(3‐[Bis‐(3‐amino‐propyl)‐amino]‐propylamino)‐N‐ditetradecylcarbamoylme‐thyl‐acetamide)/DOPE	i.v.	CIA mice	CD11b^+^ cells (macrophages and neutrophils)	Local inhibition of TNF‐α secretion and lower cPLA_2_αexpression and activity; Reduction of inflammation and cartilage damage	[221]
MCL‐1 siRNA	PLGA and PCADK microspheres loaded with HA–Chitosan NPs	i.m.	AIA rats	Macrophages	Suppressed inflammation; Reduced paw thickness	[198]
MCL‐1 siRNA; Dexamethasone	HA	i.v.	AIA mice	Macrophages	Suppressed cell infiltration and inflammation; significantly reduced pro‐inflammatory cytokines	[199]
MCL‐1 siRNA and telapamide	Hybrid NPs coated with folic acid and PEI	i.v.	AIA mice	Macrophages	Silenced MCL‐1 expression; Relieved ankle swelling and the edema of the soft tissue around the joint	[197]
NF‐κB RelA siRNA	Low‐molecular‐weight PEI–cholesterol–PEG	i.a.	CIA mice	Macrophages	Reduced NF‐κB RelA and TNF‐α mRNA level; Inhibited synovial hyperplasia, cartilage damage, lymphocyte infiltration or bone erosion; Alleviated arthritis symptoms	[187]
NF‐κB RelA siRNA; dexamethasone	PCL‐PEI and PCL‐PEG	i.v.	CIA mice	Macrophages	Inhibit the expression of NF‐kB; Reduced production of TNF‐α and IL‐1β; Macrophage repolarization from M1 to M2;	[188]
NF‐κB RelA siRNA	PCL modified with CH_2_R_4_H_2_C peptide and MPEG	i.v.	CIA mice	Synoviocytes	Suppressed NF‐κB RelA expression and reduced inflammatory cytokine levels; Prevented synovium erosion; Alleviated clinical symptoms	[204]
ERN1 siRNA	PEI and PBAA coupled with cell‐penetrating peptides and folic acid	i.v.	CIA mice	Macrophages	Reduced inflammation; Slowed RA progression; Inhibited swelling of hind paws and joints	[192]
BTK siRNA	Cationic lipid‐assisted PEG‐b‐PLGA NPs	i.v.	CIA mice	Macrophages; B cells	Inhibited BTK expression; Reduced joint inflammation and bone loss;	[194]
HnRNP A2/B1 siRNA	Cationic liposome RPR209120/DOPE	i.v.	CIA mice; K/BxN serum–transfer induced arthritis mice	Monocyte/macrophages	Reduced hnRNP A2/B1 expression; lowered IL‐23 and TNF‐α levels; Alleviated arthritic symptoms; Reduced disease incidence	[191]
PTEN mRNA	Macrophage membrane vesicles and PLGA	i.v.	CIA mice	FLSs	Activated PTEN pathway; Inhibited synovitis and joint damage	[211]
CRACM1 shRNA	Lentiviral vectors	i.a.	CIA mice	Osteoclasts	Inhibition of CRACM1; Decreased osteoclast numbers and activity; Mitigated joint inflammation, cartilage destruction, and bone deformities	[227]
TLR7 shRNA	Lentiviral vector	i.a.	CIA rats	FLSs	Downregulated TLR7 expression; Suppressed microvessel densities and VEGF levels; Reduced pro‐inflammatory cytokines; Decreased microvessel density and inhibited arthritis	[209]
Galectin‐3 shRNA	Lentiviral vector	i.a.	CIA rats	FLSs	Reduced expression of rat galectin‐3; Suppressed synovial hyperplasia, cartilage erosion, and leukocyte infiltration	[213]
miR‐21	Polymeric nanoparticles composed of poly‐l‐lysine and cis‐aconitic anhydride and conjugated with helical polypeptides	i.v.	ZIA mice	Macrophages	Suppression of PDCD4 mRNA levels; Increase in IL‐10 secretion; Balanced M1 and M2 macrophage phenotypes; Suppressed inflammatory cell infiltration; Reduced synovial thickening	[183]
miR‐23b	Fluorinated PAMAM	i.v.	AIA rats; CIA mice	FLSs; macrophages	Restored miR‐23b level; Inhibited expression of inflammatory factors (TNF‐α, IL‐1β and IL6); Suppressed inflammation and increased bone density; Ameliorated typical RA symptoms;	[215]
miR‐23b	Tetrahedral framework nucleic acids	i.p.	CIA mice	FLSs	Suppressed migration of synovial fibroblasts and levels of pro‐inflammatory and osteoclastogenic cytokines; Improved symptoms	[216]
**IBD**						
TNF‐α siRNA	Modified cyclodextrins	i.r.	DSS‐induced colitis mice	Intestinal tissue cells	Reduced TNF‐α and IL‐6; Improved clinical symptoms	[234]
TNF‐α siRNA	DOTAP and PPADT	Oral	DSS‐induced colitis mice	Macrophages and neutrophils	Diminished TNF‐α mRNA levels; Suppressed neutrophil invasion; Restored full epithelium and fossae structure	[235]
TNF‐α siRNA and IL‐22	Galactose‐functionalized NPs consisting of PLGA and chitosan	Oral	DSS‐induced colitis mice	Macrophages	Inhibited expression of TNF‐α; Accelerated mucosal healing and promoted recovery of colon tissue	[236]
TNF‐α siRNA; IP‐10 siRNA; KC siRNA	A calcium phosphate core coated with PLGA and PEI	i.r.	DSS‐induced colitis mice	Intestinal tissue cells	A specific knockdown of target genes at the site of inflammation; Ameliorated intestinal inflammation	[239]
IL‐22 mRNA	Lipid NPs consisting of lipids extracted from ginger source NPs	Oral	DSS‐induced colitis mice	Intestinal tissue cells	Enhanced IL‐22 protein expression; Accelerated healing process	[237]
CD40 ASO	Amphoteric liposomes (nov038/CD40)	i.v.	TNBS‐induced colitis mice	Macrophages	Suppressed CD40 expression; Reversed neutrophils infiltration, colon wall thickening, ulcerations, goblet cells loss, and fibrosis	[238]
CD98 siRNA	siRNA/PEI complexes coated with PLA and PVA	Oral	DSS‐induced colitis mice	Intestinal epithelial cells and macrophages	Reduced CD98 expression in mouse colonic tissues; decreased colitis	[241]
CD98 siRNA and single‐chain CD98 antibodies	PEG‐urocanic acid‐modified chitosan	Oral	CD4+CD45RBhigh T cells induced colitis Rag−/− mice; DSS‐induced colitis mice	Intestinal epithelial cells and macrophages	Reduced CD98 expression; Anti‐inflammatory and mucosal protective effects	[242]
CD98 siRNA and curcumin	HA‐functionalized NPs consisting PLGA and chitosan	Oral	DSS‐induced ulcerative colitis FVB male mice	Intestinal epithelial cells and macrophages	Inhibited CD98 and TNF‐α expression; Prevented mucosal damage and reduced inflammation; A better therapeutic effect compared to the single drug‐based formulations	[243]
Cyclin D1 siRNA	Liposomes connected with β7 integrins‐targeted antibody	i.v.	DSS‐induced colitis mice	Intestinal tissue cells	Decreased CyD1 mRNA level; Decreased TNF‐α and IL‐12 mRNA level; Reduced intestinal tissue damage, inhibited leukocyte infiltration, and reversed weight loss and hematocrit reduction	[246]
Cyclin D1 siRNA and TNF‐α siRNA	Type B gelatin NPs entrapped in a PCL microsphere	Oral	DSS‐induced colitis mice	Intestinal tissue cells	Decreased colonic levels of TNF‐α and CyD1; Suppressed expression of certain pro‐inflammatory cytokines; Weight regaining and relieved symptoms	[247]
**Psoriasis**						
TNF‐α shRNA	Lentiviral vectors	i.d.	Psoriasis xenograft transplantation mice	Skin tissue cells	Reduction in TNF‐α mRNA levels; Histological normalization of the skin structure and reduction in skin thickness	[251]
TNF‐α siRNA	glyceryl dibehenate and PAH	Topical skin application	Imiquimod‐induced psoriasis mice	Skin tissue cells	Decreased level of TNF‐α; Alleviated epidermal thickening and inhibited leukocyte infiltration	[252]
TNF‐α siRNA and tacrolimus	Nanostructured lipid carriers consisting of glycerol distearate and PEI	Topical transdermal delivery	Imiquimod‐induced psoriasis mice	Skin tissue cells	A reduction of the cytokine TNF‐α expression; Restrained psoriatic plaques and mitigated epidermal thickening	[253]
DEFB4 siRNA	Liposomes composed of DOTAP, cholesterol, sodium cholate and ethanol	Administration usum externum	Skin‐humanized mouse model for psoriasis	Skin tissue cells	Decreased hBD‐2 mRNA and protein expression; Restored transglutaminase activity, keratin expression, and epidermal appearance	[256b]
DEFB4 siRNA, TSLP siRNA and KRT17 siRNA	Liposomes composed of DOTAP, cholesterol, sodium cholate and ethanol	Topical skin application	Full‐­thickness psoriasis‐­reconstructed skin model	Skin tissue cells	Inhibited expression of target gene; Inhibited proliferation of keratinocytes	[257]
Plasmid coding IL‐4	Ultradeformable cationic liposomes consisting of DOTAP, DOPE and sodium deoxycholate	Topical transdermal delivery	Psoriasiss in the K14‐VEGF transgenic mouse model	Skin tissue cells	Effective plasmid expression; Alleviated skin lesions in diseased mice; Inhibited vascular proliferation and inflammation	[262]
NF‐κB c‐Rel siRNA	PEG‐b‐poly(l‐lysine)‐b‐poly(l‐leucine) micelles	i.p.	Imiquimod‐induced psoriasis mice	Skin tissue cells	Knockdown of the expression of NF‐κB c‐Rel; Psoriasis prevention and amelioration	[259]
NF‐κB RelA siRNA	Tetrahedral framework nucleic acids	Topical skin application	Imiquimod‐induced psoriasis mice	Keratinocytes and dendritic cells	Up to 70% delivery efficiency; Blocked NF‐κB pathway and suppressed inflammation; A significant reduction in erythema and scaling formation	[260]
EGFR siRNA	Gold nanoparticles	Topical skin application	Imiquimod‐induced psoriasis mice	T cells	EGF gene expression; Reduced epidermis thickness; Inhibited cell proliferation	[263]
miR‐197	Quaternized starch	Topical skin application	A psoriatic mouse model made by transplanting psoriatic patient‐derived uninvolved skin onto SCID mice	Keratinocytes	Decreased expression of the miR‐197 target proteins; Reduced psoriatic activity markers	[268]
**Asthma**						
Syk siRNA	DOTAP/DOPE	i.t.	OVA‐induced asthma rats	Neutrophils, eosinophils and macrophages	A significant 71% reduction in total BAL cell count in asthmatic rats; lowered TNF‐α levels; inhibitory rates of over 50% on bronchoconstriction	[281]
VDBP siRNA; dexamethasone	PEI	i.t.	OVA‐induced asthma mice	Macrophages, airway epithelial cells, and type I lung cells	A 50% decrease in VDBP mRNA levels in mouse lung tissue; Anti‐inflammatory effects; Alleviation of asthma symptoms, including excessive mucus production and airway inflammation	[283]
GATA‐3 shRNA	Lentiviral vectors	i.t.	OVA‐induced asthma mice	T cells	Decreased GATA‐3 RNA expression in lungs; Inhibited airway inflammation; Decreased eotaxin, IL‐5, and IL‐13 levels in BALF; Suppressed airway hyperresponsiveness	[285]
anti‐miR‐145 ASO	Cationic lipid NPs composed of Staramine and StaraminemPEG	i.v.	HDM‐induced asthma mice	Eosinophils, neutrophil and macrophages	Reduced miR‐145 levels; Anti‐inflammatory effects; Alleviated mucin secretion and airway obstruction	[287]
**MS**						
Lkb1 siRNA	Cationic lipid‐assisted PEG‐PLGA NPs	i.d.	EAE mice	Dendritic cells	Suppress Lkb1 expression; Sustained immunotolerant phenotype in DCs; Delayed onset and inhibited development of MS	[273]
MOG35‐55 mRNA	Cationic liposomes composed of DOTMA and DOPE	i.v.	EAE mice	T cells	Alleviated symptoms in EAE mice	[275]
Plasmid coding miR‐219	Chitosan conjugated to tragacanthic acid and glutathione	i.v.	Cuprizone‐induced demyelination mice	Oligodendrocyte precursors	An over threefold increase in miR‐219 levels; Enhanced remyelination	[277]
**SLE**						
miR‐125a	mPEG‐PLGA‐PLL NPs	i.v.	MRL/lpr mice	T cells	Increased miR‐125a levels; Alleviated lymphadenopathy and splenomegaly; Reduced skin lesions	[298]
**AS**						
BMP‐2 siRNA	PEI‐capped MnFe2O4 NPs	i.v.	Curdlan‐induced arthritis mice	hMSCs and osteoblasts	Reduced BMP‐2 levels; Alleviated local ectopic ossification	[304]
**Uveitis**						
TLR3 siRNA	pSP‐conjugated chitosan	Subretinal injections	Autoimmune uveitis mice	Retinal pigment epithelium cells	Specific inhibition of TLR3 expression; Reduced inflammatory cell infiltration; preservation of retinal structure	[294]

**Abbreviations**: hMSCs, Human mesenchymal stem cells; OVA, Ovalbumin; HDM, House dust mites; EAE, experimental autoimmune encephalomyelitis; Lkb1, Liver kinase B1; VEGF, Vascular endothelial growth factor; i.a., intraarticular; i.p., intraperitoneal; i.v., Intravenous; i.m., Intramuscular; i.r., Intrarectal; i.t., Intratracheal; i.d., Intradermal; HA, Hyaluronic acid; PEG, Poly(ethylene glycol); PEI, Polyethyleneimine; PLA, Poly(lactic acid); PVA, Polyvinyl alcohol; PLGA, Poly(lactide‐co‐glycolide); PCL, Polycaprolactone; PCADK, poly (cyclohexane‐1, 4‐diylacetone dimethylene ketal); PBAA, poly(β‐amino amine); PAH, poly(allylamine hydrochloride); PAMAM, Poly(amidoamine); PPADT, Poly‐(1,4‐phenyleneacetone dimethylene thioketal); PLL, Poly‐L‐lysine; FLS, Fibroblast‐like synoviocyte; RA, Rheumatoid arthritis; IBD, Inflammatory bowel disease; MS, Multiple sclerosis; SLE, Systemic lupus erythematosus; AS, Ankylosing spondylitis; KC, Keratinocyte‐derived cytokine; IP‐10, Interferon gamma‐induced protein 10; MCL‐1, Myeloid Cell Leukemia‐1; BSP, Betamethasone sodium phosphate; cPLA2α, Cytosolic phospholipase A2α; ERN1, Endoplasmic Reticulum to Nucleus Signaling 1; BTK, Bruton's tyrosine kinase; hnRNP, Heterogeneous nuclear ribonucleoprotein; Syk, Spleen tyrosine kinase; VDBP, Vitamin D‐binding protein; CIA, Collagen‐induced arthritis; AIA, Adjuvant‐induced arthritis; CAIA, collagen antibody‐induced arthritis; ZIA, zymosan A‐induced arthritis; DSS, Dextran sodium sulfate; TNBS, 2,4,6‐Trinitrobenzene sulphonic acid.

PEI, comprising polymerized ethyleneimine monomers, exhibits a strongly cationic nature attributed to numerous amino (NH_2_─) groups and an extensively branched molecular structure.^[^ ^121^ ^]^ These features endow PEI with superior gene condensation, proton buffering for nuclear escape, and outstanding transfection properties.^[^ ^122^ ^]^ Both the transfection efficiency and the toxicity of PEI, however, exhibit an upward trend in tandem with molecular weight.^[^ ^123^ ^]^ Strategies to mitigate PEI toxicity involve introducing hydrophobic characteristics, where achieving an optimal balance between hydrophobicity and overall positive charge is crucial for successful nucleic acid delivery. Oskuee et al. enhanced the hydrophobicity by introducing alkyl carboxyl groups to branched PEI, correlating up to 20% carboxylation with improved endosomal escape. Increasing alkyl chain length for enhanced hydrophobicity also bolstered siRNA complex stability and reduced toxicity compared to 25 kDa PEI.^[^ ^124^ ^]^ PEI derivatives with environmentally responsive characteristics can shield the positively charged surface from the adsorption of negatively charged proteins that typically induce immune reactions in the bloodstream. Zhang et al. crosslinked low molecular weight oligoethyleneimine (OEI) with sulfonyl links (TK) for selective degradation in hypoxia‐induced reactive oxygen species (ROS)‐rich environments, resulting in a stimulus‐responsive cationic water‐soluble polymer (OEI‐TKx).^[^ ^125^ ^]^ In comparison to PEI/DNA complexes, OEI‐TKx/DNA complexes demonstrated notable ROS responsiveness with reduced toxicity and better transfection efficiency.

Poly‐(lactic‐*co*‐glycolic acid) (PLGA), a copolymer of polylactic acid (PLA) and polyglycolic acid (PGA), is known for its excellent biodegradability and biocompatibility.^[^ ^126^ ^]^ PLGA undergoes hydrolytic biodegradation into its original monomers, lactic acid and glycolic acid, with the degradation rate dependent on various parameters, including molecular weight, lactide/glycolide ratio, and the shape and structure of the polymer matrix.^[^ ^127^ ^]^ To prevent the rapid clearance of intravenously injected PLGA from circulation, Fu et al. developed monomethoxy(PEG)‐PLGA‐poly(L‐lysine) (mPEG‐b‐PLGA‐b‐PLL) for the delivery of miRNA‐122 antagomir, obtaining enhanced sustained and effective delivery in vitro and in vivo; a single dose achieved 28 days of miRNA‐122 inhibition.^[^ ^128^ ^]^ PLGA‐based nanoparticles (NPs), known for their favorable safety profile, have been highly popular in nucleic acid delivery research.^[^ ^129^ ^]^ Due to the electrostatic repulsion between anionic acid groups in the PLGA polymer and the phosphate backbone of nucleic acids, the electrostatic interaction between nucleic acids and PLGA is low, and the use of PLGA NPs alone is insufficient for effective gene silencing. The introduction of cationic shaping agents, such as DOTAP, PEI, or polyamines, enhances the affinity between the PLGA matrix and nucleic acids, thereby increasing the loading capacity.^[^ ^130^ ^]^ Katas et al. utilized a spontaneous modified emulsification diffusion method to form spherical positively charged composite NPs with a weight ratio of 29:1 for PLGA and PEI, where the nitrogen‐to‐phosphorus (N/P) ratio of PEI determined the transfection efficiency of the NPs for siRNA.^[^ ^131^ ^]^

Dendritic polymers (e.g. PAMAM dendrimers, PEGylated dendrimers, and polyether‐copolyester dendrimers) constitute a class of highly branched polymers.^[^ ^132^ ^]^ They exhibit a distinctive structure consisting of three primary components: a central core, repetitive branching units, and terminal groups.^[^ ^132^ ^]^ The augmentation of repeated branching units governs the generations of dendrimers, establishing a direct correlation with dendrimer size and globular shape, where higher generations correspond to larger particle sizes. Introducing diverse terminal groups, such as PEG, signal peptides, or cell surface receptor ligands, imparts specific functionalities and properties to dendritic polymers.^[^ ^121^, ^133^ ^]^ Dong et al. engineered dendritic polymers with RGDK peptides for targeted siRNA delivery to cancer cells and achieved high cellular affinity and uptake (Figure 4).^[^ ^134^ ^]^ Sayed et al. used PAMAM‐histidine nanocarriers for miRNA (miR‐194‐5p, miR‐214‐3p, and antagomiR‐122‐5p) delivery to inhibit pro‐apoptotic genes, which prevented cardiomyocyte apoptosis and increased viability.^[^ ^135^ ^]^

Polycaprolactone (PCL) has emerged as a semi‐crystalline, biodegradable polyester synthesized by the controlled ring‐opening polymerization of 3‐caprolactone (CL). PCL and its derivatives have shown potential as non‐viral polymeric gene delivery agents, effectively regulating gene expression.^[^ ^136^ ^]^ A challenge in the application of PCL for nucleic acid drug delivery is the lack of cationic groups on the molecule for interacting with negatively charged siRNA. Therefore, researchers have chemically conjugated PCL with other polymers containing positive functional groups, thus forming cationic amphiphiles. For example, Zhu et al. developed biodegradable cationic micelles composed of poly(2‐dimethylaminoethyl methacrylate)‐PCL‐poly(2‐dimethylaminoethyl methacrylate) (PDMAEMA‐PCL‐PDMAEMA) blocks with varying molecular weights, enabling the efficient co‐delivery of siRNA and doxorubicin to cancer cells. Low molecular weight PDMAEMA‐PCL‐PDMAEMA has demonstrated superior in vitro siRNA transfection efficiency compared to the commonly used 25 kDa branched PEI (bPEI).^[^ ^137^ ^]^

#### 3.2.3 Exosomes and membrane‐based nanomaterials

Exosomes as small vesicles, typically measuring 30 to 150 nm in diameter, are known for efficiently transporting bioactive molecules like proteins, lipids, and nucleic acids across biological barriers.^[^ ^138^ ^]^ One of the important prerequisites in the development of an efficacious exosome‐based drug delivery system involves the judicious selection of the most appropriate donor cell type. Notably, exosomes derived from mesenchymal stem cells (MSCs) inherently possess anti‐fibrotic and anti‐inflammatory attributes across various tissues, including muscle, kidney, and liver.^[^ ^139^ ^]^ These properties endow them with suitability not only as platforms for nucleic acid delivery but also for harnessing their inherent therapeutic potential. Zhao et al. effectively utilized exosomes derived from subcutaneous adipose tissue‐derived MSCs (MSCs^SC^‐Exos) for miR‐199a‐3p delivery in an osteoarthritis (OA) rat model.^[^ ^140^ ^]^ This innovative approach not only mitigated the pathological severity of cartilage through the miR‐199a‐3p‐mediated mTOR autophagy pathway but also demonstrated a remarkable regenerative effect on damaged cartilage via MSCs^SC^‐Exos.

However, loading larger nucleic acid molecules into exosomes is challenging due to their small size.^[^ ^141^ ^]^ Electroporation can assist in loading short‐sequence nucleic acids like miRNA and siRNA but is less effective for mRNA and plasmid.^[^ ^142^ ^]^ As a solution, Tsai et al. pre‐incubated mRNAs with cationic lipid solutions before loading lipid‐coated mRNAs into purified exosomes through mixing and incubation.^[^ ^143^ ^]^ Munagala R et al. developed Exosome‐PEI matrices (EPM) for the co‐delivery of plasmid and siRNA^[^ ^144^ ^]^ and achieved an exceptionally high nucleic acid encapsulation rate (>90%) compared to electroporation (<5%) and ExoFect (approximately 35%).

The surface modification of exosomes with ligands can enhance targeting specificity for drug delivery in various diseases. Yan et al. modified biomimetic exosomes with compounds (folate, PEG, and cholesterol) (Figure 4) to create an actively targeted delivery carrier for dexamethasone (DEXA) sodium phosphate (FPC‐Exo/DEXA).^[^ ^145^ ^]^ Biodistribution studies have demonstrated enhanced accumulation at the sites of inflammation in arthritic mice.

In addition, functionalizing nanomaterials with cell membranes can endow nanomaterials with excellent biointerface properties.^[^ ^146^ ^]^ The cell membranes of different types of cells have complex antigen profiles and unique biological functions, breaking through the limitations of traditional nanomaterial surface modification in biomedical applications.^[^ ^147^ ^]^ Recently, nanoparticles coated with macrophage‐derived microvesicles (Figure 4) have effectively delivered immunosuppressants, such as tacrolimus, to inflamed joints in arthritic mice, showcasing its promising therapeutic potential.^[^ ^148^ ^]^

#### 3.2.4 Protein/peptide‐based vectors

Cell‐penetrating peptides (CPPs), protamine, and various other protein/peptide vehicles have been proven effective for delivering nucleic acids.^[^ ^149^ ^]^ The distinct advantage of protein/peptide carriers lies in the precise control over the composition of functional units (i.e., amino acids) within the peptides, enabling the facile and flexible synthesis of sequences with diverse functionalities. Furthermore, the size of protein/peptide particles and the encapsulation capacity for nucleic acids can be accurately regulated based on their monodispersity. CPPs, short sequences consisting of approximately 5–30 peptides, possess the ability to transgress cellular membranes, facilitating the translocation of their cargo (including proteins and nucleic acids) across a wide range of cell types. The association between nucleic acid molecules and CPPs can occur through charge‐based attraction or chemical covalent bonding. CPPs can traverse cellular membranes without inducing cellular damage and subsequently undergo degradation upon intracellular entry. Therefore, they have emerged as valuable carriers that enhance cellular uptake and intracellular release mechanisms, garnering increasing attention from researchers. Protamine, extracted from the mature gonads of fish and mammals, is an alkaline cationic peptide with a molecular weight of approximately 3800 Da, prominently rich in arginine residues.^[^ ^149^ ^]^ Its essential in vivo role involves condensing the sperm genome by bridging the arginine‐rich DNA anchoring domain with multiple phosphorylation sites on sperm cell DNA, forming stable complexes of protamine and DNA that protect the DNA from enzymatic degradation. Capitalizing on the inherent characteristics of protamine, researchers often combine it with other materials (e.g. lipids) to form composite carriers for efficient nucleic acid delivery.^[^ ^150^ ^]^ Protamine is typically incorporated as the innermost layer in these complex carriers, taking charge of nucleic acid condensation and protection. Given its wide availability, affordability, and facile complexation, protamine has gained significant traction as a versatile nucleic acid delivery platform.

#### 3.2.5 Inorganic nanoparticles

Inorganic nanoparticles are synthesized by combining inorganic particles, biodegradable polycations, and common inorganic materials such as gold, silica, and carbon.^[^ ^151^ ^]^ Among INPs, extensive research has been conducted on gold nanoparticles (AuNPs) (Figure 4) in the fields of inorganic chemistry and nanomaterials.^[^ ^151^ ^]^ AuNPs exhibit chemical inertness and display non‐toxic monodisperse nanostructures, making them an ideal choice for various ligand functionalizations.^[^ ^151^ ^]^ However, the inherent inertness of AuNPs hinders their timely metabolism, resulting in a prolonged half‐life that limits their clinical utility. Copper sulfide complexation has been explored as a means to improve this aspect.^[^ ^152^ ^]^ Amorphous silicon dioxide nanoparticles (SiNPs), composed of silicon dioxide, have emerged as versatile drug delivery carriers due to their facile manufacturing processes and excellent biocompatibility.^[^ ^153^ ^]^ These SiNPs, whether non‐porous (solid) or mesoporous, have been extensively investigated for their ability to deliver small molecule drugs, therapeutic proteins, and nucleic acid molecules.^[^ ^153^ ^]^ Non‐porous SiNPs feature a straightforward fabrication process, while mesoporous SiNPs (Figure 4) are highly regarded for their pronounced cargo‐loading capacity, facile surface modification, and efficient biodegradation.^[^ ^154^ ^]^ Nucleic acid molecules can be immobilized onto the positively charged surface of SiNPs or encapsulated within a silica shell to evade enzymatic degradation. Furthermore, emerging inorganic materials such as carbon nanotubes (CNTs) and metal‐organic framework materials (MOFs) have garnered significant attention.^[^ ^155^ ^]^

### 3.3 Passive and active targeting

In order to achieve targeted drug delivery, the design principles of nanoparticle formulations can be categorized into two main groups: passive targeting and active targeting.^[^ ^156^ ^]^ The former is defined as the preferential accumulation of nano‐carriers at sites of injury, infection, and inflammation.^[^ ^157^ ^]^ This phenomenon is attributed to endothelial cell dysfunction and impaired lymphatic drainage leading to abnormal vascular networks, pathologies commonly observed in cancer.^[^ ^157^ ^]^ Passive targeting relies on adjusting the physical properties of NPs, such as size, shape, hardness, and surface charge.^[^ ^158^ ^]^ Ren et al. adjusted four parameters of liposomes (surface charge, size, PEG chain length, and PEG concentration) and implemented liposome‐encapsulated DEXA for RA treatment.^[^ ^159^ ^]^ The results showed that liposomes with a diameter of 100 nm, those carrying a slight negative charge (15.07 ± 0.85 mV) and incorporating 10% 5 kDa PEG exhibited the highest inflammatory joint targeting capability. Dilliard et al. achieved selective organ targeting by incorporating the fifth SORT lipid into conventional LNPs.^[^ ^160^ ^]^ By introducing SORT molecules to alter the apparent acid dissociation constant (pKa), liver‐targeting SORT LNPs were found to have a pKa in the range of 6–7, while that of spleen‐targeting LNPs ranged from 2–6.

On the other hand, active targeting involves delivering therapeutic drugs to pathological sites via molecular recognition while avoiding healthy cells or tissues.^[^ ^161^ ^]^ Active targeting entails the surface modification of NPs with chemical or biological moieties that specifically bind to receptors or other cellular features highly expressed in the target organ cells.^[^ ^162^ ^]^ Seong et al. developed bone‐targeting peptide (ASPSERSER)_6_‐conjugated liposomes, and tissue biopsy following the intravenous injection of these Cy5‐labeled liposomes revealed predominant retention in the long bones of mice.^[^ ^163^ ^]^ Considering the expression of folate receptors in RA synovial macrophages and their increased affinity for folate binding,^[^ ^164^ ^]^ folic acid‐modified chitosan NPs have been explored for the targeted delivery of nucleic acid drugs in RA therapy.^[^ ^165^ ^]^ Coco et al. engineered three types of nanoparticles: Eudragit S100‐coated ovalbumin NPs, *N,N,N*‐trimethylchitosan chloride‐coated ovalbumin NPs, and mannose‐modified ovalbumin NPs. Among them, only the latter NPs successfully traversed the Caco‐2 intestinal barrier via endocytosis, demonstrating significant accumulation in inflamed colon sites ex vivo.^[^ ^166^ ^]^

## 4 THERAPEUTIC APPLICATIONS OF NUCLEIC ACID DELIVERY IN IMIDS

Immune dysfunction and excessive inflammation are common themes in the development and progression of various IMIDs. These intricate effects result in varying degrees of tissue and organ damage, leading to corresponding symptoms. At the cellular and molecular levels, aberrant activities of immune and inflammatory cells, such as macrophages, neutrophils, and dendritic cells, along with dysregulation of relevant cytokines (such as TNF‐α, IL‐1β, and NF‐κB) or receptor functions, set the stage for these diseases. As tools for regulating gene expression, nucleic acid molecules target the upstream genes of these molecules, coordinating their expression to influence and restore normal cellular physiological activities. Simultaneously, the design of appropriate, targeted carriers enables stable delivery of these nucleic acid molecules, thereby offering favorable and promising therapeutic effects.

### 4.1 Rheumatoid arthritis (RA)

**FIGURE 5:**
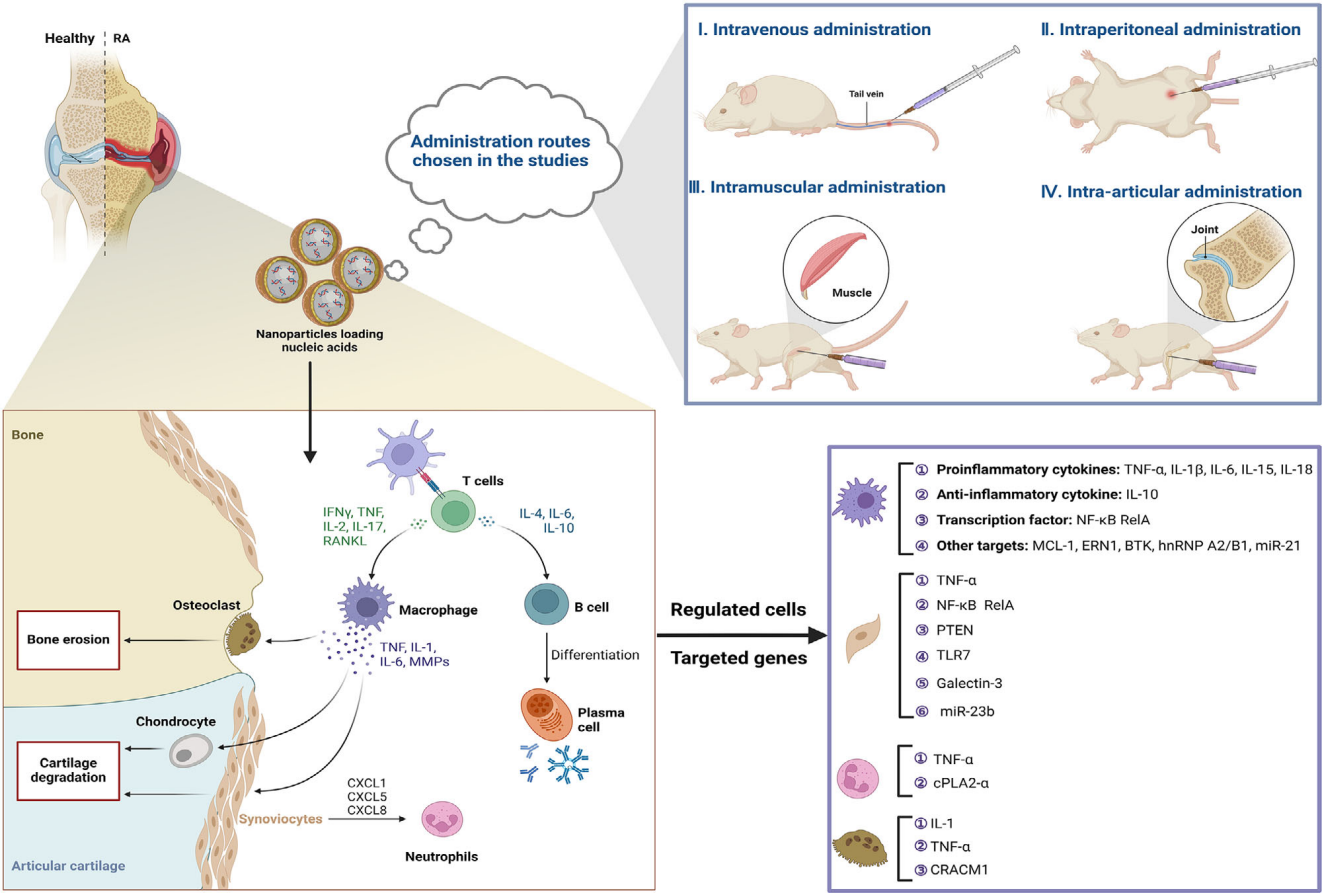
In rheumatoid arthritis (RA), nucleic acid drugs are commonly used to regulate macrophages, synovial cells, neutrophils, and osteoclasts to exert corresponding therapeutic effects. Simultaneously, various modes of drug administration are adopted.

RA is a complex systemic autoimmune inflammatory disease that predominantly affects joints in the wrists, fingers, ankles, and toes.^[^ ^167^ ^]^ Affecting 0.5–1.0% of the global population, it presents clinical symptoms such as joint swelling, severe pain, stiffness, synovitis, and cartilage damage, leading to long‐term physical and psychological problems, including chronic pain and irreversible joint damage causing disability.^[^ ^168^ ^]^ Treatment approaches for RA primarily involve the administration of medications like non‐steroidal anti‐inflammatory drugs, glucocorticoids, and disease‐modifying antirheumatic drugs (DMARDs). However, these may elicit severe adverse reactions, including gastric ulcers, hypertension, hepatotoxicity, and renal abnormalities.^[^ ^169^ ^]^ Currently, nucleic acid‐based therapy is a novel strategy with the advantage of precision and specificity, that can precisely target the occurrence and progression of arthritis by modulating individual molecules or signaling pathways (Figure 5, Table 3).

#### 4.1.1 Inhibition of pro‐inflammatory effects on macrophages

Macrophages are important players in the progression of RA. The presence of macrophage infiltration in synovial tissue serves as an early indicator of RA, and its level is positively correlated with the severity of joint erosion. Macrophages also significantly contribute to this condition by secreting a variety of inflammatory mediators, including TNF‐α, IL‐1β, and IL‐6, orchestrating a critical role in the cytokine network that drives synovial inflammation.^[^ ^170^ ^]^ Furthermore, macrophages exhibit the ability to facilitate endothelial cell chemotaxis and proliferation, promoting angiogenesis and the infiltration of inflammatory cells.^[^ ^171^ ^]^ Therefore, modulating macrophages via nucleic acid delivery is a promising avenue for RA treatment as it can reduce the inflammatory response and hinder disease progression.

Inhibiting the secretion of pro‐inflammatory cytokines by macrophages via gene therapy is one of the most widely adopted strategies. TNF‐α intensifies interactions between macrophages and synovial fibroblasts, T cells and B cells, ultimately driving synovial inflammation and joint damage.^[^ ^172^ ^]^ Song et al. utilized a PEI‐based carrier to deliver plasmid encoding TNF‐α shRNA, effectively reducing cellular the TNF‐α mRNA level to 23% in macrophages compared to that of the control group in vitro.^[^ ^173^ ^]^ As indicated by immunohistochemistry staining, plasmid encoding TNF‐α shRNA effectively inhibited TNF‐α in collagen‐induced arthritis (CIA) mice, significantly alleviating the progression of RA. Howard et al. employed CSs for TNF‐α siRNA delivery, resulting in an approximately 66% knockdown of TNF‐α in primary peritoneal macrophages in vitro.^[^ ^174^ ^]^ Subsequent intraperitoneal injection into CIA mice achieved a TNF‐α knockdown of approximately 44%, leading to the alleviation of mouse joint swelling. Previous studies on downregulating TNF‐α in macrophages through siRNA delivery have also demonstrated that the targeted regulation of TNF‐α in macrophages can significantly alleviate inflammatory symptoms and delay the progression of RA.^[^ ^175^ ^]^ In addition, it also seems worthwhile to regulate other pro‐inflammatory factors secreted by macrophages through nucleic acid delivery. Recognizing the crucial role of IL‐1β in fostering bone erosion and cartilage degradation through the upregulation of matrix metalloproteinase (MMP).^[^ ^176^ ^]^ Song et al. developed lipid‐polymer hybrid NPs (FS14‐NP) to efficiently deliver small interfering RNA targeting IL‐1β (siIL‐1β) to macrophages.^[^ ^177^ ^]^ The intravenous administration of FS14‐NP/siRNA yielded the rapid accumulation of siIL within macrophages in the joints of collagen antibody‐induced arthritis (CAIA) mice, as detected by immunohistochemical staining, leading to diminished IL‐1β, TNF‐α, MMP‐3, and MMP‐13, accompanied by a significant alleviation of bone erosion and cartilage destruction. Khoury et al. delved into a triple siRNA lipoplex therapy aimed at inhibiting pro‐inflammatory cytokines, specifically targeting IL‐1β, IL‐6, and IL‐18.^[^ ^178^ ^]^ As detected by enzyme‐linked immunosorbent assay (ELISA), triple siRNA lipoplexes significantly and effectively suppressed targeted pro‐inflammatory factors in mice knee joints, achieving an impressive 80% macrophage transfection rate, implying prolonged and superior disease relief. Due to the pleiotropic pro‐inflammatory effects of IL‐15,^[^ ^179^ ^]^ Zhang et al. developed PEI‐based IL‐15 receptor β chain (IL‐15Rβ) siRNA composite NPs, effectively silencing IL‐15Rβ.^[^ ^180^ ^]^ Weekly intravenous injections of IL‐15Rβ siRNA/PEI NPs led to a significant TNF‐α and IL‐1β reduction in the ankle joints of adjuvant‐induced arthritis (AIA) rats, providing substantial relief from clinical arthritis symptoms after 3 weeks, along with a notable reduction in synovial tissue inflammation and cartilage/bone damage.

Increasing the secretion of anti‐inflammatory cytokines (such as IL‐10) from macrophages can suppress the inflammatory response, aiding in the regulation of immune system overactivity. In a study conducted by Jain et al., non‐aggregated alginate‐based NPs were designed to selectively target macrophages for the delivery of plasmid encoding IL‐10.^[^ ^181^ ^]^ The polymerase chain reaction (PCR) results showed that IL‐10 plasmid NPs effectively transformed macrophage phenotypes in arthritic rat joints from pro‐inflammatory M1 (approximately 15%) to anti‐inflammatory M2 (approximately 66%), alleviating features of arthritis such as inflammatory cell infiltration, heightened osteoclast activity and bone resorption. Li et al. encapsulated IL‐10 plasmid and betamethasone sodium phosphate (BSP) into exosomes derived from M2‐type macrophages (M2 Exo) to achieve a combined therapeutic effect.^[^ ^182^ ^]^ Beyond elevating IL‐10 mRNA levels and promoting the M2 polarization of macrophages, the administration of M2 Exo/IL‐10 plasmid, as demonstrated by Hematoxylin and Eosin (H&E) stained tissue sections, yielded a homogeneous distribution of mouse joint cartilage cells, accompanied by comparatively reduced inflammatory cell infiltration and cartilage erosion. To harness the inhibitory role of miR‐21 on programmed cell death protein 4 (PDCD4), an IL‐10 inhibitor, Deng et al. designed polymer NPs conjugated with transmembrane helical peptides for the co‐delivery of miR‐21 and IL‐4. ^[^ ^183^ ^]^ In vitro studies showed that these NPs achieved a 93% suppression of PDCD4 mRNA levels in RAW 264.7 cells stimulated with lipopolysaccharide, leading to a 2.9‐fold increase in IL‐10 secretion and effective inhibition of pro‐inflammatory cytokines like TNF‐α. In zymosan A‐induced arthritis (ZIA) mice, intravenous injection of miR‐21/IL‐4 NPs balanced the M1 and M2 macrophage phenotypes, suppressed inflammatory cell infiltration, and reduced synovial thickening, effectively alleviating arthritis symptoms.

Members of the nuclear factor‐kappa B (NF‐κB) transcription factor family, including NF‐κB1, NF‐κB2, RelA, RelB, and c‐Rel, play critical roles in mediating the transcription of target genes involved in immunity and inflammation.^[^ ^184^ ^]^ The pro‐inflammatory function of NF‐κB in macrophages has been well documented, which involves transforming macrophages from M2 to M1^[^ ^185^ ^]^ and promoting the secretion of various inflammatory factors, including TNF‐α, IL‐1β and IL‐6.^[^ ^186^ ^]^ Consequently, inhibiting the NF‐κB signaling pathway to modulate the pro‐inflammatory effects of macrophages represents a promising therapeutic strategy. Chen et al. developed low molecular weight PEI‐based micelles (LPCE) to deliver NF‐κB RelA siRNA (siRelA), significantly downregulating NF‐κB RelA mRNA and TNF‐α mRNA levels and promoting M1‐to‐M2 macrophage polarization.^[^ ^187^ ^]^ The intraarticular administration of siRelA/LPCE inhibited synovial hyperplasia, cartilage damage, lymphocyte infiltration and bone erosion. Wang et al. further combined siRelA with DEXA, which more strongly inhibited the activation of NF‐κB signaling pathway and the secretion of pro‐inflammatory cytokines (TNF‐α and IL‐1β).^[^ ^188^ ^]^

Autoantibodies against heterogeneous nuclear ribonucleoprotein (hnRNP) A2/B1, which has emerged as a pivotal coactivator for the transcription factors implicated in proinflammatory pathways,^[^ ^189^ ^]^ have been detected in approximately in 20–30% of RA patients.^[^ ^190^ ^]^ To suppress this process, Herman et al. employed liposomes for delivering hnRNP A2/B1 siRNA.^[^ ^191^ ^]^ RT‐qPCR and ELISA analysis revealed that hnRNP A2/B1 siRNA/lipoplexes effectively reduced hnRNP A2/B1 protein and mRNA levels (up to 50%) but also efficiently dampened IL‐23 and TNF‐α levels in monocytes/macrophages, resulting in mitigated inflammation, bone erosion and arthritis symptoms, as well as a decreased incidence of arthritis.

Certain enzymes encoded by genes can promote the release of inflammatory factors in macrophages, such as inositol‐requiring enzyme 1 alpha (IRE1α) that is encoded by the endoplasmic reticulum to nucleus signaling 1 (ERN1) gene.^[^ ^192^ ^]^ Feng et al. designed folate receptor‐targeted NPs based on PEI and delivered ERN1 siRNA (siERN1), thus alleviating hind paw swelling (averaged at 2.81 mm compared to 4.07 mm in the control group) in CIA mice, accompanied by the disappearance of symptoms such as bone loss and signs of cartilage erosion.^[^ ^192^ ^]^ Previous studies further demonstrated that the activation of Bruton's tyrosine kinase (BTK) can promote the polarization of pro‐inflammatory macrophages and the production of pro‐inflammatory cytokines.^[^ ^193^ ^]^ Based on this mechanism, Zhao et al. used cationic lipid‐assisted NPs (CLAN) to package BTK siRNA (CLAN_siBTK_) for the efficient delivery of siRNA to macrophages and B cells,^[^ ^194^ ^]^ thus inhibiting BTK (up to 84% reduction) to reduce ankle joint thickness and bone loss in arthritic mice.

MCL‐1, a member of the anti‐apoptotic B‐cell lymphoma‐2 (Bcl‐2) protein family, is notably upregulated in the synovial macrophages of RA patients.^[^ ^195^ ^]^ MCL‐1 shields overactivated macrophages from apoptosis by suppressing apoptotic signaling pathways.^[^ ^196^ ^]^ Based on the above mechanism, Li et al. employed PEI‐based NPs fortified with photosensitizers for co‐delivering the chemotherapeutic drug tirapazamine and MCL‐1 siRNA, and this strategy effectively silenced MCL‐1 in macrophages.^[^ ^197^ ^]^ The combined effects of MCL‐1 siRNA‐mediated RNA interference, photodynamic therapy and tirapazamine hypoxia‐activated chemotherapy led to a reduction in ankle joint swelling and erythema, markedly alleviating arthritis inflammation in AIA rats, as demonstrated by the H&E staining results. Other studies have also indicated that single MCL‐1 siRNA delivery^[^ ^198^ ^]^ or combined administration with DEXA^[^ ^199^ ^]^ organized chondrocyte alignment, reduced inflammatory cell infiltration and significantly alleviated symptoms such as joint swelling, erythema and soft tissue edema.

In summary, macrophages, as important sources of pro‐inflammatory cytokines, are primarily regulated in the context of anti‐inflammatory actions. The most commonly employed strategy involves directly inhibiting pro‐inflammatory cytokines or enhancing the expression of anti‐inflammatory factors using nucleic acid drugs. However, in complex disease environments, solely inhibiting a specific cytokine may have limited effectiveness. Therefore, alternative approaches include targeting multiple pro‐inflammatory cytokines or upstream molecules and signaling pathways involved in cytokine synthesis and secretion (such as NF‐κB), thereby exerting multiple anti‐inflammatory effects.

#### 4.1.2 Harmonization of invasive fibroblast‐like synoviocytes

Fibroblast‐like synoviocytes (FLSs) play a pivotal role in maintaining normal physiological functions in synovial tissue, which encompass constructing the synovial lining, secreting synovial fluid and proteins into the joint, and providing plasma proteins to adjacent cartilage and the joint cavity.^[^ ^200^ ^]^ However, under the inflammatory conditions of RA, FLSs undergo a transition from benign mesenchymal cells to destructive and invasive tumor‐like cells, characterized by diminished sensitivity to apoptosis and the overexpression of adhesion molecules.^[^ ^201^ ^]^

Inflammatory FLSs have the capacity to generate an array of pro‐inflammatory cytokines, such as IL‐1 and TNF‐α, further stimulating the production of destructive enzymes by synovial cells. Chen et al. devised biomimetic nanoparticles encapsulating TNF‐α and IL‐6 siRNA (M@P‐siRNAsT/I), demonstrating effective targeting of both FLSs and macrophages in CIA.^[^ ^202^ ^]^ Apart from effectively downregulating TNF‐α and IL‐6 expression and mitigating FLS invasiveness, M@P‐siRNAsT/I supressed bone erosion, cartilage damage and immune cell infiltration, significantly alleviating symptoms such as synovial fibrosis, synovial hyperplasia.

NF‐κB activation has been detected in the synovial tissue of RA patients, which preceded the development of clinical symptoms and intensified with disease progression.^[^ ^203^ ^]^ Kanazawa et al. engineered polymer‐based micelles for the delivery of siRelA to synovial cells, leading to the suppression of RelA mRNA,^[^ ^204^ ^]^ which markedly reduced hind paw thickness and clinical scores in CIA mice.

Toll‐like receptors (TLRs) constitute a membrane protein family and their activation is associated with the production of pro‐inflammatory cytokines and the activation of different immune cell populations.^[^ ^205^ ^]^ The arthritis‐inducing capability of TLR ligands has been demonstrated in animal experiments,^[^ ^206^ ^]^ and TLR‐specific antagonists successfully alleviated CIA.^[^ ^207^ ^]^ Given the abundant expression of TLR7 in the synovial intimal and subintimal regions of RA joints,^[^ ^208^ ^]^ Chen et al. investigated the therapeutic effects of lentivirally delivered TLR7 shRNA (Lt.shTLR7) in CIA rats.^[^ ^209^ ^]^ The intraarticular injection of Lt.shTLR7 effectively suppressed TLR7 in FLSs and reduced vascular endothelial growth factor (VEGF) and pro‐inflammatory cytokines, resulting in decreased microvessel density (approximately 60% of the control group) and the inhibition of arthritis.

PTEN represents a pivotal therapeutic target for alleviating RA symptoms.^[^ ^210^ ^]^ Chen et al. utilized a macrophage membrane carrier overexpressing TNF‐α receptors to deliver PTEN mRNA (MR@P‐mPTEN), with the objective of elevating PTEN protein levels in FLSs.^[^ ^211^ ^]^ MR@P‐mPTEN exerted a dual action by competitively binding TNF‐α and activating the PTEN pathway, ultimately leading to the inhibition of synovitis and joint damage.

Galectin‐3 (Gal3) has been reported not only to participate in inflammation but also to contribute to the activation of FLSs in RA.^[^ ^212^ ^]^ To inhibit Gal3, Wang et al. intraarticularly injected lentivirus‐loaded Gal3 shRNA into the joints of CIA rats, suppressing synovial hyperplasia, cartilage erosion, and leukocyte infiltration, thereby improving arthritis symptoms.^[^ ^213^ ^]^

MiR‐23b holds a potential in balancing the effects of cells like FLSs in the inflamed synovium by modulating the NF‐κB signaling pathway.^[^ ^214^ ^]^ Han et al. employed fluorinated polyamidoamine (FP) for miR‐23b delivery, which was nonspecifically taken up by FLSs in vivo and resulted in the restoration of miR‐23b levels in the synovium along with a significant reduction in inflammatory factors (TNF‐α, IL‐1β, and IL‐6).^[^ ^215^ ^]^ Treatment with FP/miR‐23b suppressed inflammation and increased bone density (FP/miR‐23b group, 5994.66 mg/cc vs untreated group, 5362.3 mg/cc) in AIA mice, ameliorating typical RA symptoms. Wang et al. utilized tetrahedral framework nucleic acids (tFNAs) to enhance the stability of miR‐23b for efficient delivery.^[^ ^216^ ^]^ Intraperitoneal injection of tFNAs‐miR‐23b complex effectively suppressed the migration of synovial fibroblasts, as well as the synthesis and secretion of pro‐inflammatory and osteoclastogenic cytokines, thereby improving the symptoms associated with the disease.

In RA, invasive FLSs also contribute to pro‐inflammatory effects. The selection of various nucleic acid targets involved directly targeting inflammatory factors or indirectly regulating the production or function of inflammatory factors through targeting upstream molecules. These strategies help suppress synovitis and joint damage, alleviating arthritis symptoms.

#### 4.1.3 Suppression of pro‐inflammatory effects on neutrophils

In RA, neutrophils present heightened abundance in inflamed areas, secreting large amounts of cytokines and other immune mediators.^[^ ^217^ ^]^ Excessive neutrophil recruitment contributes to the establishment of chronic inflammatory states and consequent tissue damage at the joint sites.^[^ ^218^ ^]^ Consequently, timely intervention in neutrophil function and activity has emerged as an attractive and efficient approach for RA treatment.

Komano et al. developed TNF‐α siRNA‐loaded NPs (TNF‐α siRNA/WS) primarily regulating neutrophils within the joints of CIA mice^[^ ^219^ ^]^ to yield approximately 50% reduction in TNF‐α mRNA levels in vivo, thereby reducing arthritis incidence by 55% and significantly lowering clinical arthritis scores.

Previous studies have explored the systemic blockade of cytosolic phospholipase A2‐alpha (cPLA_2_α), inhibiting arthritis and bone destruction by reducing neutrophil recruitment to inflammatory sites and suppressing Th1‐type cytokine production.^[^ ^220^ ^]^ Courties et al. devised cationic lipid‐coated lipid complexes for delivering cPLA_2_α siRNA, thereby modulating the effects of neutrophils.^[^ ^221^ ^]^ Weekly treatment with cPLA2α siRNA lipoplex reduced cPLA2α mRNA levels to near‐normal levels, resulting in a significant reduction in leukocyte infiltration and synovial inflammation, with an almost 60% reduction in inflammatory areas.

For neutrophils, the regulation of their inflammatory effects by nucleic acid drugs targeting inflammatory factors also limits their infiltration and recruitment to inflammatory sites. As a result, these nucleic acid‐based therapies effectively alleviate joint inflammation and damage.

#### 4.1.4 Regulation of osteoclast activity

Osteoclasts and osteoblasts play central roles in maintaining skeletal homeostasis. In RA, persistent joint inflammation heightens osteoclast differentiation and activity while hindering osteoblast differentiation and function, resulting in an imbalance of bone homeostasis and subsequent bone degradation.^[^ ^222^ ^]^ It has been demonstrated that countering IL‐1 with monoclonal antibodies or inhibiting soluble IL‐1 type II receptors can substantially mitigate cartilage and bone damage.^[^ ^223^ ^]^ Fernandes et al. devised chitosan‐based NPs for the delivery of plasmids encoding the IL‐1 receptor antagonist (IL‐1Ra), effectively facilitating IL‐1Ra expression both in vitro and in vivo.^[^ ^165b^ ^]^ AIA rats treated with IL‐1Ra plasmid NPs exhibited a reversal of inflammatory bone turnover and a diminished number of osteoclasts (> 50%) in the bone marrow cavity.

TNF‐α can induce RANKL expression, stimulate the binding of RANKL to its receptor RANK, mediate osteoclast proliferation and differentiation, and impede osteoblast differentiation and bone formation.^[^ ^224^ ^]^ Shi et al. utilized modified chitosan‐based nanocarriers for the delivery of TNF‐α siRNA, thus decreasing TNF‐α levels by approximately 30% in the knee joints of arthritic mice.^[^ ^165a^ ^]^ The number of bone marrow interstitial osteoclasts decreased to one‐third that of the control group, with cortical bone density in the tibia increasing by more than twofold and trabecular thickness returning to normal levels.

Ca^2+^ release–activated channels (CRACs) facilitate osteoclast proliferation and promote osteoclast fusion, enabling them to possess multiple nuclei for functional efficacy.^[^ ^225^ ^]^ To partially block CRACs, Liu et al. intraperitoneally injected lentiviral vectors carrying the CRAC homolog modulator 3 (CRACM3) shRNA (Lt.sh CRACM3) in CIA mice, which resulted in reduced osteoclast numbers (74% of the control group) and activity (76% of the control group).^[^ ^226^ ^]^ Subsequently, this research team was able to overcome the adverse effects associated with systemic administration and opted for the intraarticular injection of CRACM1 shRNA (Lt.sh CRACM1), suppressing CRACM1 especially in the synovium.^[^ ^227^ ^]^ Intraarticular Lt.sh CRACM1 treatment significantly decreased osteoclast numbers (47.27%) and activity (19.21%), along with reduced pro‐inflammatory cytokines and inflammatory cell infiltration, thereby mitigating joint inflammation, cartilage destruction and bone deformities.

In the inflammatory state of RA, receptor‐enhanced osteoclasts undergo proliferation and enhanced differentiation under the stimulation of inflammatory factors, leading to imbalances in bone homeostasis and bone destruction. Therefore, targeting inflammatory factors such as TNF‐α or related signal receptors to inhibit osteoclast activity, coordinate bone formation, and control the osteoclast‐to‐osteoblast ratio can have anti‐inflammatory and bone‐protective effects. On the other hand, blocking cell factors (e.g. IL‐1) that promote cartilage and bone destruction is also a desirable method to maintain bone and osteoclast balance.

### 4.2 Inflammatory bowel disease (IBD)

**FIGURE 6:**
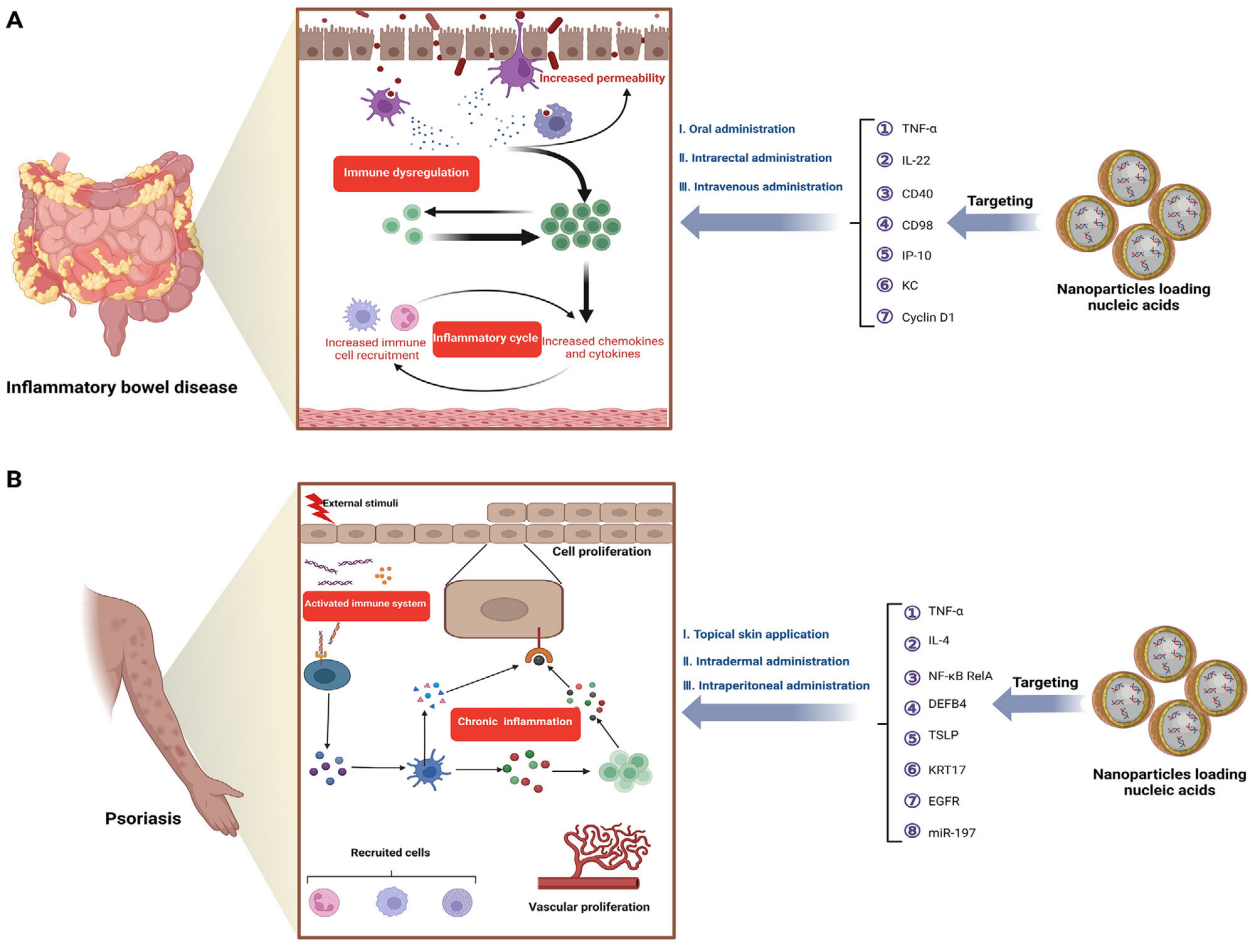
(A) Immune and inflammatory dysregulation in the gut in inflammatory bowel disease and the currently investigated therapeutic targets. (B) Localized dysregulated responses and pathological cell proliferation in psoriatic skin, along with the currently investigated therapeutic targets.

IBD is a chronic condition characterized by idiopathic inflammation of the gastrointestinal tract (Figure 6A), with the most prevalent clinical forms being ulcerative colitis and Crohn's disease.^[^ ^228^ ^]^ IBD patients endure a spectrum of debilitating symptoms, including fatigue, abdominal pain, weight loss, and diarrhea, impacting approximately 0.3% of the global population and imposing a substantial economic burden on society.^[^ ^229^ ^]^ Drug therapy is fundamental in addressing IBD, with essential medications like 5‐aminosalicylic acid derivatives, corticosteroids and immunosuppressants such as azathioprine and 6‐mercaptopurine.^[^ ^230^ ^]^ Regrettably, however, it is imperative to recognize that the associated side effects are challenging to avoid. These encompass gastrointestinal symptoms, infection risks, and notably, the potential for leukopenia, in addition to possible repercussions on liver and kidney function.^[^ ^230^, ^231^ ^]^ Nucleic acid therapies targeting disease‐related molecules offer novel therapeutic avenues in this field (Table 3).

TNF‐α initiates and promotes the inflammatory response in IBD, leading to the apoptosis of intestinal epithelial cells and exacerbating tissue damage.^[^ ^232^ ^]^ Anti‐TNF‐α therapy has demonstrated good efficacy in treating IBD.^[^ ^233^ ^]^ On this basis, McCarthy et al. administered TNF‐α siRNA to mice with dextran sulfate sodium (DSS)‐induced colitis using modified CD.^[^ ^234^ ^]^ CD‐TNF‐α siRNA treatment effectively silenced TNF‐α mRNA (73 ± 13%) and IL‐6 (58 ± 19%) mRNA in proximal colon tissues, and improved the clinical symptoms, including weight gain and reduced rectal bleeding. To achieve targeted delivery to inflamed colonic sites, Wilson et al. incorporated poly‐(1,4‐phenyleneacetone dimethylene thioketal) (PPADT), responsive to ROS, into the lipid carrier for TNF‐α siRNA delivery, leading to the restoration of colonic epithelial integrity and reduced neutrophil invasion.^[^ ^235^ ^]^ Xiao et al. co‐delivered TNF‐α siRNA and IL‐22 (a healing‐promoting cytokine), addressing inflammation and promoting mucosal healing simultaneously.^[^ ^236^ ^]^ The application of LNPs to deliver IL‐22 mRNA increased IL‐22 protein in the colonic mucosa of mice, promoting the accelerated healing of DSS‐induced colitis.^[^ ^237^ ^]^ Notably, Arranz et al. systemically delivered amphoteric liposomes carrying CD40 (a member of the TNF superfamily) ASOs, which effectively suppressed experimental colitis without inducing immunosuppression.^[^ ^238^ ^]^ Frede et al. detected significantly elevated levels of TNF‐α, interferon gamma‐induced protein 10 (IP‐10), and keratinocyte‐derived cytokine (KC) proteins in colonic tissues of colitis mice.^[^ ^239^ ^]^ The researchers then targeted these three inflammatory mediators and designed calcium phosphate (CaP)/PLGA NPs carrying siRNAs against them.^[^ ^239^ ^]^ Through efficient delivery to intestinal epithelial cells (IECs) and immune cells in DSS‐induced colitis mice, triple siRNA CaP/PLGA NPs suppressed TNF‐α mRNA by 40%, which led to KC and IP‐10 mRNA decreasing by 50% and reduced disease activity scores.

CD98, a type II transmembrane protein, has crucial importance in both macrophage activation and the progression of intestinal inflammation.^[^ ^240^ ^]^ Xiao et al. synthesized PEI‐based NPs for CD98 siRNA delivery, predominantly silencing CD98 in IECs and macrophages.^[^ ^241^ ^]^ Histological staining demonstrated inhibited neutrophil infiltration and the overall restoration of crypt and villus structures, affirming the anti‐inflammatory effects of these NPs. Furthermore, this research group explored chitosan‐based NPs modified with CD98‐targeting antibodies for delivering CD98 siRNA, resulting in an approximately 50% increase in nanoparticle uptake in vitro.^[^ ^242^ ^]^ Besides, the co‐delivery of CD98 siRNA and natural products (e.g. curcumin^[^ ^243^ ^]^ and berberine^[^ ^244^ ^]^) demonstrated superior anti‐inflammatory effects and enhanced mucosal healing compared to the sole delivery of CD98 siRNA.

Cell cycle protein D1 (CyD1) regulates cell cycle progression from the G1 phase to the S phase.^[^ ^245^ ^]^ In the context of IBD, CyD1 exhibits abnormal upregulation within IECs and immune cells. Focusing on CyD1 as a potential therapeutic target, Peer et al. devised lipid‐based NPs, known as β7I‐tsNPs, for the precise delivery of CyD1 siRNA to colonic mononuclear cells, effectively normalizing CyD1 mRNA levels and concomitantly suppressing TNF‐α and IL‐12.^[^ ^246^ ^]^ These NPs substantially ameliorated colonic tissue damage in colitis mice, proficiently curbing white blood cell infiltration and eliciting a reversal of both weight loss and reduced blood cell hematocrit levels. Kriegel et al. harnessed a polymer‐based carrier system termed NIMOS for the co‐delivery of CyD1 siRNA and TNF‐α siRNA.^[^ ^247^ ^]^ This dual siRNA delivery approach successfully reduced colonic TNF‐α and CyD1 levels, subsequently inhibiting specific pro‐inflammatory cytokines (IL‐1α and ‐β, interferon‐λ) and ultimately resulting in weight gain and alleviating symptoms in the colitis mouse model.

The selection of nucleic acid targets for IBD is mostly based on the aberrant expression of various inflammatory factors and their upstream molecules. The effect cells regulated by nucleic acids include IECs and resident mucosal immune cells. Additionally, considering the important barrier function of the intestinal mucosa, the delivery of certain cell factors and drugs that promote mucosal healing is also an important consideration. Furthermore, as an important digestive organ, when delivering therapeutic nucleic acids to the intestine, the choice of suitable carriers and administration methods needs to be further considered.

### 4.3 Psoriasis

Psoriasis is a chronic autoimmune skin disorder affecting approximately 2–3% of the global population.^[^ ^248^ ^]^ It presents with recurrent red patches on various body parts, such as the scalp, knees, elbows, palms, and soles, covered by thick, white scales.^[^ ^249^ ^]^ The pathogenesis of psoriasis involves complex interactions between genetic susceptibility, environmental triggers (sun exposure, infections, stress, etc.), and both innate and adaptive immune components. Current treatment modalities for psoriasis encompass topical medications (corticosteroids and vitamin D analogs), oral systemic immunosuppressants (e.g. methotrexate, vitamin A analogs, and cyclosporine), and phototherapy. Nevertheless, these therapies lack universal effectiveness and are linked to broad immunosuppression and/or organ toxicities, posing challenges when administered over the long term.^[^ ^250^ ^]^ On the other hand, nucleic acid delivery therapies offer the potential to silence aberrant gene expression, harmonizing the skin's immune and inflammatory responses, and restraining excessive cell growth (Figure 6B), thereby alleviating disease symptoms (Table 3).

In psoriatic skin lesions, elevated TNF‐α levels drive disease progression by interacting with other cytokines. TNF‐α inhibitors not only decrease the population of immune and inflammatory cells in the skin but also curb epidermal hyperplasia, ameliorating the disease symptoms. Jakobsen et al. utilized lentiviral vectors to deliver TNF‐α shRNA (Lt.sh TNF‐α) to mice transplanted with human psoriatic skin.^[^ ^251^ ^]^ The intradermal injection of Lt.sh TNF‐α resulted in a 29% reduction in TNF‐α mRNA levels within the graft, leading to histological normalization of the skin structure and reduced skin thickness. Suzuki et al. delivered TNF‐α siRNA using the photodynamic internalization (PCI) strategy, achieving twice the delivery efficiency and effectively decreasing TNF‐α levels in imiquimod (IMQ)‐induced psoriasis mice.^[^ ^252^ ^]^ To augment the inhibitory outcomes, Viegas et al. employed a nanostructured lipid carrier (NLC) for the co‐delivery of TNF‐α siRNA and the immunosuppressant tacrolimus (TAC), resulting in a sevenfold TNF‐α reduction within the skin tissue of psoriatic mice, surpassing the efficacy of TNF‐α siRNA alone.^[^ ^253^ ^]^ Beyond notable anti‐inflammatory effects, the localized application of TNF‐α siRNA‐TAC‐NLC effectively restrained psoriatic plaques and mitigated epidermal thickening.

Human β‐defensin‐2 (hBD‐2), encoded by the DEFB4 gene, was initially isolated from scales of psoriatic skin^[^ ^254^ ^]^ and stands out as the most psoriasis‐specific protein,^[^ ^255^ ^]^ making it a promising target for psoriasis treatment. Lambert et al. employed cationic lipid‐based nanovesicles to deliver DEFB4 siRNA to a humanized mouse model of psoriatic skin.^[^ ^256^ ^]^ Localized treatment reinstated markers of epidermal proliferation and differentiation (transglutaminase activity, keratin) and restored the appearance of the stratum corneum to levels akin to normally regenerating human skin. Furthermore, the group targeted three upregulated genes in psoriasis (KRT17, DEFB4, and TSLP) for siRNA delivery, leading to reduced specific psoriasis markers (e.g. involucrin, keratin 16, and Ki‐67) and inhibiting keratinocyte proliferation in a full‐thickness bovine psoriasis‐reconstructed skin model.^[^ ^257^ ^]^

Due to the significant adverse effects associated with drugs targeting the entire NF‐κB family,^[^ ^258^ ^]^ Fan et al. employed polymer micelles for the targeted delivery of siRNA to specifically inhibit NF‐κB c‐Rel (sicRel).^[^ ^259^ ^]^ This strategy effectively reduced c‐Rel protein and interrupted the expression of various inflammatory cytokines (IL‐1β, IL‐6, IL‐17, and IL‐23) in psoriasis mouse skin. The intraperitoneal injection of sicRel/micelleplex effectively mitigated skin inflammation and thickening in psoriasis, as evidenced by H&E‐stained skin sections. In a study by Zhang et al., tFNA s with exceptional transdermal capabilities were employed for the targeted delivery of siRelA.^[^ ^260^ ^]^ In vitro experiments demonstrated that tFNAs‐siRelA achieved delivery efficiencies of more than 70% to both HaCaT cells and dendritic cells (DCs). This efficient delivery system successfully blocked the NF‐κB pathway and effectively suppressed inflammation. In IMQ‐induced psoriasis mice, the localized administration of tFNAs‐siRelA exhibited substantial therapeutic efficacy against psoriasis, resulting in a significant reduction in erythema and scaling formation.

Therapies based on the anti‐inflammatory cytokine IL‐4 have shown favorable tolerability and effectiveness in psoriasis treatment.^[^ ^261^ ^]^ Building on this avenue, Li et al. designed a super‐deformable cationic liposome (UCL) for the transdermal delivery of IL‐4 plasmid in a psoriasis mouse model.^[^ ^262^ ^]^ Histological examinations observed only mild epidermal hyperplasia, minimal reticular ridges on the ear surfaces, and a slight increase in tissue thickness in the dermal chambers after UCL/IL‐4 treatment. Besides, pathological scores and immunohistochemical analyses provided additional evidence for countering psoriasis and inhibiting angiogenesis and inflammation.

Epidermal growth factor (EGF) is secreted by macrophages, fibroblasts, and platelets, and it acts through paracrine signaling on epidermal cells. By binding to its receptors (EGFR), EGF plays a vital role in regulating cell growth, survival, proliferation, and differentiation of various cell types. In order to counteract abnormal cell proliferation, Nemati et al. designed AuNPs conjugated with EGFR siRNA to effectively silence the EGF gene.^[^ ^263^ ^]^ In IMQ‐induced psoriasis mice, these AuNPs successfully suppressed the epidermal thickening and functional activity of disease‐related T cells, thereby providing relief for psoriasis.

Previous studies have suggested that miR‐197 can attenuate the expression of critical receptor subunits for IL‐22 and IL‐17 (IL‐22RA1^[^ ^264^ ^]^ and IL‐17RA^[^ ^265^ ^]^), inhibiting their roles in driving psoriatic inflammation through mediating interactions between the immune system and epidermal keratinocytes.^[^ ^266^ ^]^ Reduced miR‐197 may also be linked to decreased keratinocyte proliferation and differentiation.^[^ ^267^ ^]^ To restore the level of miR‐197, Lifshiz et al. harnessed Q‐starch as its delivery vehicle (Q‐starch/miR‐197).^[^ ^268^ ^]^ As demonstrated by immunohistochemistry, the administration of this vehicle effectively suppressed IL‐22RA1 and IL‐17RA, accompanied by a notable decrease in Ki‐67 staining, indicating inhibited basal cell proliferation in psoriatic lesions.

Psoriasis is often associated with abnormal epidermal cell proliferation and elevated levels of pro‐inflammatory cytokines, which are closely related to its characteristic plaques and scaling. Therefore, nucleic acid delivery usually targets cell factors such as TNF‐α or molecules and receptors related to proliferation and differentiation. Additionally, since these nucleic acid drugs are typically used topically on the skin and need to reach the inner layers of the skin, the transdermal permeability needs to be carefully considered when designing carriers.

### 4.4 Multiple sclerosis (MS)

**FIGURE 7:**
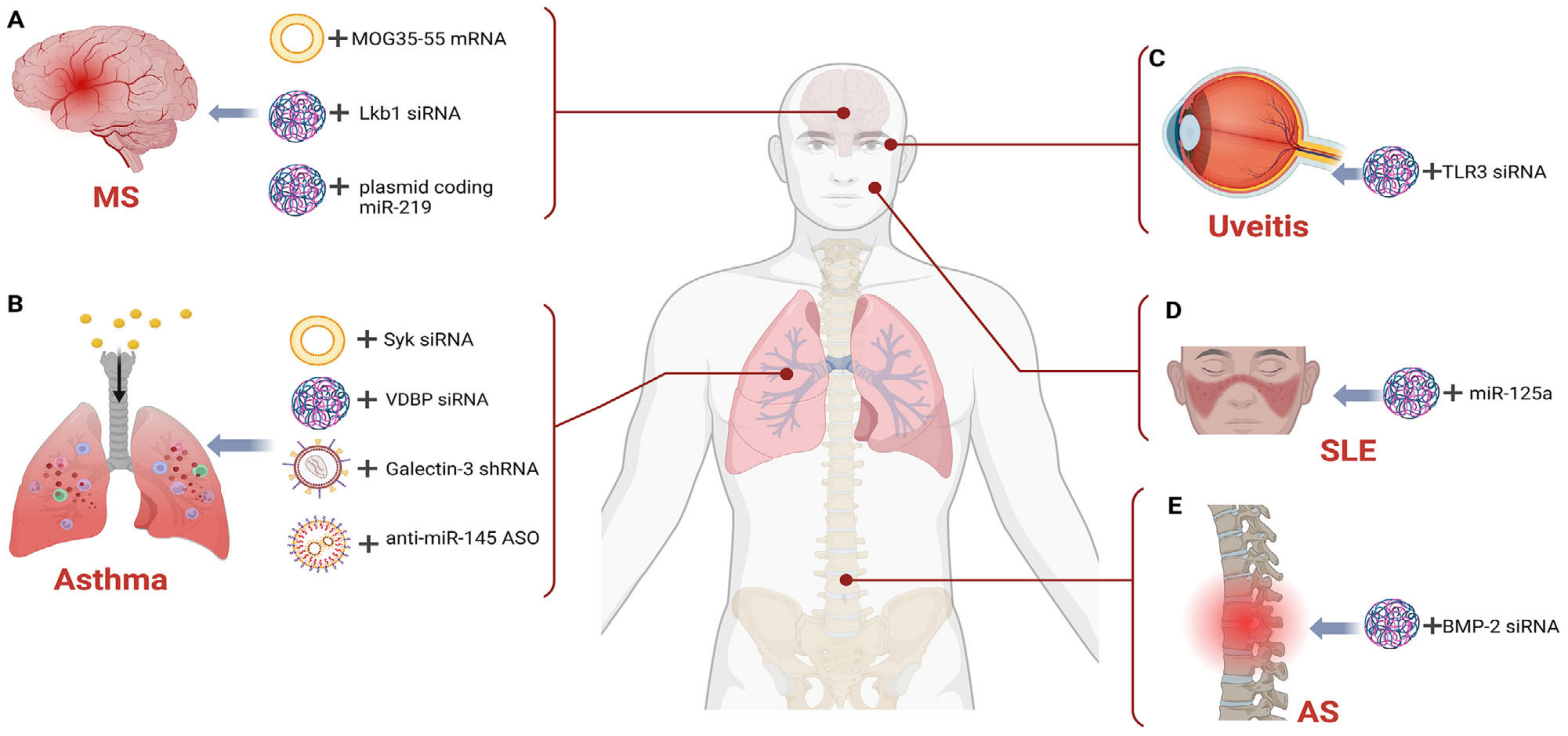
Therapeutic nucleic acids and corresponding delivery systems used for asthma, multiple sclerosis (MS), systemic lupus erythematosus (SLE), ankylosing spondylitis (AS), and uveitis. Specifically, certain diseases (e.g., SLE) typically involve multiple systems. The diseases depicted in this image are identified based on their characteristic symptoms or the primary affected organ.

Multiple sclerosis (MS) is a chronic inflammatory, demyelinating, and neurodegenerative disease of the central nervous system (CNS). It affects approximately 2.3 million people worldwide and is a leading cause of non‐traumatic disability among young individuals.^[^ ^269^ ^]^ MS is a complex, multifactorial, and immune‐mediated disease that arises from intricate gene‐environment interactions. Pathologically, MS is characterized by multifocal demyelinating lesions, inflammatory infiltration (including macrophages, T cells, and B cells), axonal damage, and loss of oligodendrocytes.^[^ ^270^ ^]^ The limited efficacy and undesirable side effects associated with current immunosuppressive therapies underline the unmet needs in MS treatment.^[^ ^271^ ^]^ However, the advancements in precise nucleic acid delivery have brought about new hope and perspectives for the treatment of this condition (Figure 7A).

Liver kinase B1 (Lkb1), a central regulatory factor for DCs metabolism and growth, plays a crucial role in maintaining immune homeostasis by balancing the responses of pro‐inflammatory T cells and regulatory T cells.^[^ ^272^ ^]^ Liu et al. utilized cationic lipid‐assisted PEG‐PLGA NPs to deliver Lkb1 siRNA to DCs in the draining lymph nodes for controlling the progression of MS in a mouse model (experimental autoimmune encephalomyelitis [EAE]).^[^ ^273^ ^]^ In vitro experiments demonstrated that NP‐mediated delivery of Lkb1 siRNA could suppress Lkb1 and sustain an immunotolerant phenotype in DCs. In vivo fluorescence imaging exhibited the accumulation of NPs in mouse lymph nodes up to 85%, thereby effectively delaying the onset and inhibiting the development of MS. Intriguingly, Krienke et al. identified a partial sequence of myelin oligodendrocyte glycoprotein (MOG_35‐55_) as a causative self‐antigen in the EAE model of MS.^[^ ^274^ ^]^ This discovery suggests that inducing immune tolerance specific to MOG_35‐55_ forms the basis for EAE therapy. This concept was also demonstrated in the experiment by Verma et al., in which MOG_35‐55_ peptide was delivered to induce immune tolerance.^[^ ^231^ ^]^ Then, Krienke et al. further employed cationic liposomes to selectively deliver mRNA translating the MOG_35‐55_ epitope to T cells, inducing tolerance and effectively alleviating symptoms in EAE mice.^[^ ^275^ ^]^ miR‐219 plays an indispensable role in the differentiation and maturation of oligodendrocyte precursors, thus facilitating axonal remyelination.^[^ ^276^ ^]^ However, previous studies have indicated downregulation of microRNA‐219a‐5p (miR‐219) in demyelinating lesions.^[^ ^276^ ^]^ To address this, Shamaeizadeh et al. employed a CS‐based carrier to deliver a plasmid expressing miR‐219 to the CNS in a cuprizone‐induced demyelination model.^[^ ^277^ ^]^ Intravenous administration of these NPs resulted in an over threefold increase in CNS miR‐219 levels compared to the control group, thereby enhancing remyelination.

In summary, nucleic acid drugs primarily induce immune tolerance or enhance remyelination in MS by regulating the expression of key molecules. However, considering the presence of the blood‐brain barrier, designing carriers that efficiently deliver nucleic acid molecules to the CNS requires careful consideration.

### 4.5 Asthma

Asthma is an IMID of global concern, affecting 339 million people worldwide and resulting in approximately 1,000 deaths per day (globalasthmareport.org Global Asthma Report 2018). This condition not only places a significant burden of disability and mortality on individuals but also poses substantial socio‐economic challenges.^[^ ^278^ ^]^ Environmental pollutants and allergens are the primary contributors to allergic sensitization and the development of asthma. While most asthma patients can manage their symptoms, including excessive mucus production, pulmonary inflammation and airway hyperresponsiveness (AHR), by using corticosteroids and β−2 adrenergic agonists, individuals with severe asthma often exhibit resistance to conventional treatments.^[^ ^279^ ^]^ Therefore, the exploration of novel, precision‐targeted therapeutic approaches for this patient group is of paramount importance (Figure 7B).

The pivotal involvement of the protein tyrosine kinase Syk in immune cell activation, lymphocyte development and the release of allergic mediators has sparked considerable interest in the formulation of Syk inhibition‐based therapies.^[^ ^280^ ^]^ Stenton et al. utilized a lipid‐based carrier to deliver Syk ASO to the lungs of rats with ovalbumin (OVA)‐induced allergic asthma, which led to a 71% reduction in the total cell count in bronchoalveolar lavage (BAL), with neutrophil counts decreasing by 74%, eosinophil counts by 85.2% and macrophages returning to normal levels.^[^ ^281^ ^]^ Effective doses of Syk ASO demonstrated robust anti‐inflammatory effects and inhibited tracheal constriction by more than 50%.

The vitamin D‐binding protein (VDBP) is primarily produced by alveolar macrophages and the bronchial epithelium. BAL VDBP concentrations correlate with increased airway inflammation, and anti‐VDBP antibody treatment can reduce airway inflammation in asthmatic mice.^[^ ^282^ ^]^ Therefore, Choi et al. employed a PEI‐based carrier for the co‐delivery of VDBP siRNA and DEXA, effectively targeting alveolar macrophages, airway epithelial cells and type I lung cells, as confirmed by the fluorescence results using cy3‐labeled siRNA and cellular binding assays.^[^ ^283^ ^]^ Alongside the substantial VDBP silencing effects (up to 50%), histological sections unveiled a significant reduction in immune cell infiltration and mucus secretion.

The transcription factor GATA‐3 regulates the release of IL‐4, IL‐5 and IL‐13 from helper T 2 cells (Th2), promoting Th2 recruitment and the activation of eosinophils, leading to airway inflammation and impaired lung function.^[^ ^284^ ^]^ To investigate the role of silencing GATA‐3 in asthma treatment, Lee et al. intratracheally administered lentiviral vectors carrying GATA‐3 shRNA (Lt.sh GATA‐3) to asthmatic mice, resulting in a significant reduction of over 50% in GATA‐3 expression in their lungs.^[^ ^285^ ^]^ Lt.sh GATA‐3 also decreased eosinophil and lymphocyte infiltration and reduced the levels of inflammatory factors (IL‐5 and IL‐13), thereby suppressing airway inflammation and inhibiting the development of AHR.

MiR‐145 has a pro‐inflammatory role in the development of allergic airway inflammation induced by house dust mites (HDM), and blocking its function significantly reduces the severity of inflammatory lesions and AHR.^[^ ^286^ ^]^ On this basis, Ramelli et al. utilized PEGylated cationic lipid NPs for the delivery of anti‐miR‐145 ASO, which resulted in a marked decrease in miR‐145 levels within the lung tissues of HDM‐sensitized mice, as demonstrated by QRT‐PCR.^[^ ^287^ ^]^ A decline in BAL eosinophil and neutrophil counts, coupled with a diminished macrophage density observed in lung tissue sections, underscored the potent anti‐inflammatory effects. Concurrently, the decrease in PAS staining across the 100 to 600 mm range in mouse airways implied a reduction in mucus secretion and relief from small airway obstruction.

In asthma, the upregulation of various molecules leads to the release of allergic mediators and the recruitment of immune and inflammatory cells, resulting in airway inflammation and AHR. Therefore, the targeted inhibition of these molecules using nucleic acid drugs can effectively exert anti‐inflammatory effects and suppress airway constriction. Furthermore, the administration of nucleic acids through the airways provides additional convenience.

### 4.6 Uveitis

Uveitis, an autoimmune ocular disease involving the ciliary body, vitreous, choroid, and retina, is marked by recurrent inflammation of the retina and uvea.^[^ ^288^ ^]^ With an incidence of 24.9 cases per 100,000 individuals, uveitis stands as a major cause of blindness, contributing to 10% of all cases.^[^ ^288^, ^289^ ^]^ Subsequent to diagnosis, conventional treatments rely on systemic and local corticosteroids, either independently or in conjunction with immunosuppressive agents, aiming to avert irreversible vision loss.^[^ ^290^ ^]^ However, the combined use of corticosteroids and immunosuppressive drugs has been linked to diverse side effects and exhibits limited efficacy.^[^ ^291^ ^]^ Investigating the potential underlying pathological mechanisms is imperative to formulate targeted, safe, and efficacious strategies for uveitis treatment. Retinal pigment epithelial (RPE) cells possess the capability to trigger distinct functions, such as pathogen clearance, effector T‐cell activation or immune response suppression, by discerning various signals through specialized surface receptors (pattern recognition receptors). Toll‐like receptor 3 (TLR3) is assumed to have a pivotal role not only in stimulating the expression of pro‐inflammatory cytokines and chemokines in RPE cells^[^ ^292^ ^]^ but also in the reactivation of effector T cells and the pathogenesis of autoimmune diseases.^[^ ^293^ ^]^ To this end, Chen et al. developed phosphorylatable short peptide‐conjugated CS (pSP‐CS) for the targeted delivery of TLR3 siRNA (Figure 7C), specifically inhibiting TLR3 in RPE cells in vitro.^[^ ^294^ ^]^ The administration of pSP‐CS/TLR3‐siRNA resulted in reduced inflammatory cell infiltration and preserved retinal structure, as revealed by H&E staining, which led to a decrease in the severity of uveitis.

### 4.7 Systemic lupus erythematosus (SLE)

SLE is a condition possibly resulting in multi‐organ damage and potentially life‐threatening consequences, affecting approximately 0.02% to 0.07% of the population.^[^ ^295^ ^]^ While non‐specific immunosuppressants or anti‐inflammatory drugs like glucocorticoids, cyclophosphamide, and methotrexate are commonly used therapeutics for SLE patients, their efficacy can be inconsistent, and their prolonged use may lead to severe side effects.^[^ ^296^ ^]^ Hence, there is a pressing need to explore more efficient and safer treatment alternatives. A fundamental mechanism in SLE pathogenesis is the imbalance between effector T cells and Tregs.^[^ ^297^ ^]^ MiR‐125a deficiency can impair the immunoregulatory capacity of Tregs, while miR‐125a overexpression can stabilize Treg‐mediated self‐tolerance.^[^ ^297^ ^]^ To this end, Zhang et al. employed polymer‐based NPs to deliver miR‐125a (Figure 7D), thereby elevating its levels in T cells and effectively promoting Treg differentiation in vitro.^[^ ^298^ ^]^ Following treatment with these miR‐125a NPs, the pathological features (lymphadenopathy and splenomegaly) and clinical symptoms (skin lesions and proteinuria) in SLE model mice were significantly alleviated, with markedly decreasing serum levels of anti‐dsDNA antibodies and ANAs.

### 4.8 Ankylosing spondylitis (AS)

Approximately 0.1‐0.5% of the global population is affected by AS, a condition characterized by the inflammation of the axial skeleton and sacroiliac joints.^[^ ^299^ ^]^ Genetic factors play a significant role in most AS cases.^[^ ^300^ ^]^ Early diagnosis and timely treatment are crucial for managing AS, with treatment primarily relying on traditional medications that suppress immune responses and alleviate inflammatory symptoms.^[^ ^301^ ^]^ However, these drugs can only mitigate the inflammatory response and associated symptoms but cannot fully reverse the ossification processes caused by AS.^[^ ^301^ ^]^ Human mesenchymal stem cells (hMSCs) possess potent immunomodulatory and osteogenic differentiation capabilities, contributing significantly to bone metabolism and immune homeostasis.^[^ ^302^ ^]^ High expression of BMP‐2 has been shown to enhance the osteogenic differentiation potential of hMSCs.^[^ ^303^ ^]^ Therefore, Zheng et al. developed CH6 ligand‐modified NPs actively delivering BMP‐2 siRNA (Figure 7E) into hMSCs and osteoblasts, which contributed to a significant reduction of approximately 50% in BMP‐2 levels in inflammation‐induced hMSCs.^[^ ^304^ ^]^ The systematic delivery of BMP‐2 siRNA alleviated abnormal ossification within the ankle joints of AS model mice.

## 5 CONCLUSION AND FUTURE PERSPECTIVES

IMIDs are often characterized by immune dysregulation and chronic inflammation. Nucleic acid therapy has emerged as a novel strategy, offering precision and specificity in targeting the onset and progression of diseases by modulating individual molecules or signaling pathways. Nucleic acid drugs with the potential to treat diseases at their fundamental ‘source’ distinguish them from conventional drugs. However, they also confront a myriad of challenges. Unmodified nucleic acids are impeded from entering cells due to their intrinsic instability, abbreviated half‐life, vulnerability to endogenous nucleases, renal filtration and subsequent urinary excretion, suboptimal cellular uptake efficiency, and immunogenicity. These factors severely circumscribe the clinical applicability of nucleic acid therapeutics. To surmount these limitations and enhance nucleic acid delivery efficacy, chemical modifications and various delivery systems have emerged as common strategies. Ideally, nucleic acid delivery systems should exhibit favorable attributes encompassing exceptional biocompatibility, protracted circulation within the biological milieu, outstanding targeting capabilities, augmented cellular uptake, and efficacious escape from endosomes upon internalization. Viral vectors, as the earliest nucleic acid delivery vehicles, are fraught with limitations, including challenges in large‐scale production, potential immunogenicity, and mutagenicity, rendering them less preferable for intracellular delivery. In contrast, non‐viral carriers, particularly nanocarriers, present themselves as auspicious alternatives due to their commendable biocompatibility, diminished immunogenicity, and scalability.

This review provides an overview of the application and research pertaining to therapeutic nucleic acid delivery in IMIDs. Among the diverse classes of nucleic acids, siRNA stands out as the most extensively utilized, potentially owing to its simple molecular structure and relatively advanced manufacturing process. siRNA has been employed to inhibit various pro‐inflammatory cytokines, including TNF‐α, suppressing inflammation, and achieving therapeutic effects. Similar functions were observed for miRNA in many IMIDs diseases. For instance, in RA, miR‐23b improved typical RA symptoms and increased bone density by inhibiting inflammation. In addition, in psoriasis, miR‐197 reduced the expression of target proteins and decreased psoriasis activity markers. Nevertheless, despite their high market anticipation, ASOs have limited studies in IMIDs treatment. In addition to small nucleic acid drugs, mRNAs have also found applications. In IBD, the IL‐22 mRNA actively upregulates IL‐22, accelerating intestinal healing. This suggests the feasibility and clinical application value of the therapeutic approach of upregulating anti‐inflammatory cytokines through mRNAs in IMIDs treatment.

Delivery systems for nucleic acid drugs have been extensively researched for a wide range of conditions, including tumors, infectious diseases, metabolic disorders, immune‐related, and inflammatory diseases.^[^ ^149^, ^305^ ^]^ Lipid‐based and polymer‐based delivery systems are currently among the most mainstream and widely applied carrier systems. ^[^ ^306^ ^]^ Although there may be some overlap in the delivery vehicles used for various diseases, we have observed that the specific details and strategies for designing delivery systems in IMIDs may vary depending on the specific disease and affected site (Table 3). Firstly, IMIDs typically involve chronic inflammation in specific tissues or organs. This chronic inflammation is driven by dysregulated immune responses and can lead to tissue damage and dysfunction. Therefore, delivery vehicles used for IMIDs must possess target specificity to selectively deliver therapeutic agents to these inflamed areas while minimizing exposure to healthy tissues. Targeted delivery can be achieved through various strategies, such as utilizing ligand‐receptor interactions, antibody‐mediated targeting, or exploiting the unique features of the inflamed tissue microenvironment. Additionally, certain microenvironmental characteristics of the inflamed tissue, such as acidic pH or elevated levels of specific enzymes, can also be exploited for targeted drug delivery. pH‐sensitive or enzyme‐responsive delivery systems can be designed to release the therapeutic agent specifically in response to the altered microenvironment of the inflamed tissue, further enhancing the therapeutic effect while reducing off‐target effects. Secondly, the delivery system needs to efficiently deliver therapeutic nucleic acids to specific immune and inflammatory cells that play a crucial role in the pathogenesis of IMIDs. For example, in RA, macrophages and FLSs are the major effector cells driving chronic inflammation and joint destruction. Therefore, the delivery vehicles should be designed to facilitate the uptake and intracellular delivery of nucleic acids into these specific cell types. This can be achieved by incorporating cell‐penetrating peptides or by optimizing the physicochemical properties of the delivery system to promote cellular uptake and endosomal escape. Furthermore, the route of administration is an important consideration in the design of delivery systems for IMIDs. Different IMIDs may require different routes of administration based on the affected site and the desired therapeutic effect. For example, IBD primarily affects the gastrointestinal tract, and oral administration is often preferred. In this case, the delivery system should be designed to protect the therapeutic agent from degradation in the harsh gastric environment and facilitate its release and absorption in the intestine. Similarly, for cutaneous manifestations of IMIDs such as psoriasis, topical administration is commonly used. Thus, the delivery system needs to be formulated to ensure effective penetration of the skin and localized delivery of the therapeutic agent. Lastly, some IMIDs require long‐term or repeated treatment to maintain their therapeutic effect. In these cases, the delivery vehicle can be designed to provide sustained release of the therapeutic agent, ensuring a constant and controlled drug release over an extended period. This sustained release approach can reduce the dosing frequency and improve patient compliance. Additionally, alternative administration routes that allow for convenient and patient‐friendly drug delivery, such as inhalation or oral delivery, can also be considered for certain IMIDs.

Nucleic acid therapeutics have flourished in recent years. Currently, there are over 3000 clinical trials for nucleic acid therapeutics, and several have been approved including eight ASOs, four siRNA drugs, and two mRNA vaccines.^[^ ^307^ ^]^ With the successive approval of nucleic acid drugs in various rare and infectious disease fields, people have high hopes for breakthroughs in the field of IMIDs. After more than a decade of global clinical research and development, some drugs have reached Phase I to Phase III clinical trials and have demonstrated variable degrees of efficacy in preclinical and clinical experiments. For example, SB010 has been engineered to modulate GATA‐3 activity by disrupting mRNA expression. Results from a recently released Phase 2a clinical trial showed that inhalation of SB010 significantly reduced asthmatic reactions after allergen exposure in individuals with allergic asthma.^[^ ^308^ ^]^ Additionally, SB010 effectively reduced the Th2‐driven inflammatory response, as evidenced by decreased levels of IL‐5 and reduced sputum eosinophil counts. SB012 has entered Phase IIa clinical trials for the treatment of active ulcerative colitis (clinicaltrials.gov NCT02129439). Furthermore, a plasmid drug encoding the TNF‐α inhibitor, pEYS606, has shown significant reduction in ocular inflammation and protection of photoreceptors in preclinical experiments for the treatment of non‐infectious uveitis. It is now advancing into Phase I/II clinical trials.^[^ ^309^ ^]^ TAKC‐02, an ASO that inhibits the synthesis of MEX3B, has undergone safety evaluation for the treatment of severe asthma (clinicaltrials.gov NCT05018533).

However, the further development of nucleic acid‐based therapeutics targeting IMIDs to achieve clinical translation still faces significant challenges. The complexity of IMIDs' pathogenesis, involving dysregulation in multiple molecules and signaling pathways, adds to the difficulty. While currently approved nucleic acid drugs have demonstrated strong target specificity and significant therapeutic efficacy by primarily targeting a single disease mechanism or a single gene, their efficacy in suppressing the disease progression of IMIDs may be limited due to the multifaceted nature of these disorders. Therefore, exploring the combination of nucleic acid drugs with other therapies for treating IMIDs is a promising research direction. Additionally, it's crucial to consider the heterogeneity of IMIDs patients. IMIDs encompass over 80 diseases, and patients can exist in different stages of disease progression with varying severity. This diversity poses further challenges in developing effective nucleic acid drugs. Thus, tailoring treatment approaches to the individual characteristics and disease stage of each patient will be crucial for maximizing the therapeutic benefits of nucleic acid therapies in IMIDs. Furthermore, while research efforts have mainly focused on common types of IMIDs (e.g. RA, IBD and psoriasis), there is a growing recognition of the importance of understanding the underlying mechanisms of ‘immunity’ and ‘inflammation’ in a broader context. Advances in our understanding of the immune system and the complex interplay of inflammatory processes are illuminating the potential applications of nucleic acid therapies beyond the common types of IMIDs. These emerging insights offer exciting opportunities for expanding the use of nucleic acid‐based therapeutics to address a wider range of IMIDs, bridging existing knowledge gaps and improving patient care.

## CONFLICT OF INTEREST STATEMENT

The authors declare no conflicts of interest.
